# Herbivore Identity and Species Richness Shape Fruit Chemical and Quality Responses to Foliar Herbivory

**DOI:** 10.1002/ece3.73970

**Published:** 2026-07-07

**Authors:** Xavier Ozowara, Taylor M. Sloey, Susan R. Whitehead

**Affiliations:** ^1^ Department of Biological Sciences Virginia Polytechnic Institute and State University Blacksburg Virginia USA; ^2^ Department of Biological Sciences Old Dominion University Norfolk Virginia USA

**Keywords:** foliar herbivory, induced defenses, insect herbivory, phenolics, specialized metabolism

## Abstract

The cascading consequences of foliar herbivory on fruit chemistry and quality remain relatively underexplored, with most existing studies focusing on the effects of only one or two attackers. In this study, we examine how increasing herbivore species richness during foliar herbivory alters strawberry (*Fragaria* × *ananassa* Duch) fruit quality and phenolic chemistry. We conducted a greenhouse experiment with single species feeding and different combinations of feeding by three generalist lepidopteran species: *Spodoptera frugiperda, Heliothis virescens, Helicoverpa zea*. Species fed on leaves for 72 h as fruits ripened, and fully ripe fruits were harvested 72 h after feeding for quality and phenolic analysis. We tested three hypotheses: (1) foliar herbivory induces complex changes in fruit traits, including specialized chemistry (phenolic content, richness, and composition) and quality (fruit weight, sugar content, and pH); (2) these changes may vary depending on the species of foliar herbivore; and (3) attack from multiple herbivores leads to more intense and complex changes, including higher diversity of specialized metabolites. We found that foliar herbivory, regardless of herbivore identity, decreased fruit pH and increased phenolic richness. Other effects of herbivory varied depending on the identity of the herbivore. Notably, *Spodoptera frugiperda* induced greater changes in soluble sugar content and flavanol, flavan‐3‐ols, and benzoic acid content and richness. We observed a directional decrease in average fruit weight in response to increased species richness during foliar herbivory. *Synthesis*: Plant responses to foliar herbivory are highly variable and context dependent. We found that foliar herbivory by combinations of three generalist species induced distinct changes in fruit chemistry and quality. This study provides a key insight into the complexity of plant‐herbivore interactions, with implications for broader ecosystem function.

## Graphical abstract

The cascading effects of foliar herbivory on specialized metabolism, specifically within reproductive tissue like fruit, are relatively understudied. We conducted a greenhouse experiment with varying combinations of three common pest species on fruiting strawberry plants. We found strong effects of herbivore identity on fruit quality and chemistry as well as a decrease in fruit weight when more insect pests fed on plants.

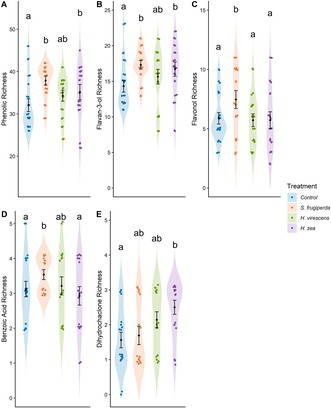

## 1 Introduction

Herbivory is among the most detrimental interactions plants experience, as the regeneration of lost tissue requires a substantial amount of energy (Strauss and Irwin 2004). To mitigate these pressures, plants have evolved a sophisticated toolkit of specialized metabolites, which primarily serve to mediate interactions, including deterring and countering the effects of herbivory (Strauss and Zangerl 2002). Chemical defenses in plant tissue can be constitutive (maintained at baseline levels regardless of attack) or induced (upregulated following an attack) (Gatehouse 2002). Induced defenses are highly plastic, with wide temporal and spatial variability in induction after the occurrence of herbivory (Heil 2010; Gatehouse 2002; Wang et al. 2015). Specialized metabolites responsive to herbivory are primarily regulated via the jasmonic acid (JA) and salicylic acid (SA) pathways (Fernández De Bobadilla et al. 2022; Walling 2000). These pathways regulate plant responses to different feeding strategies; the JA pathway is primarily activated by chewing damage, whereas the SA pathway often responds to piercing herbivores and pathogens (Eisenring et al. 2018; Moran and Thompson 2001). The effective activation of these pathways depends on a plant's ability to detect chemical cues left behind by herbivores, a complex task given the extensive variability in how, when, and where herbivores feed on plants (Wetzel et al. 2023).

While feeding, herbivores leave behind a cocktail of molecules called herbivore‐associated elicitors (HAEs). HAEs can be found in the oral secretions, fecal matter, and egg depositions on the plant surface and enter plant cells via damage to the epidermis (Bonaventure et al. 2011; Malook et al. 2022). These chemicals can be highly specific to the individual species or broadly possessed by the larger taxonomic group (Bonaventure et al. 2011). During herbivory, receptor kinases found in the plasma membrane of plant cells detect HAEs released during herbivory and respond with an upregulation of chemical defenses (Malook et al. 2022). With certain HAEs being species‐specific, some plants have evolved to recognize the unique chemical signature and tailor defensive responses to the herbivore identity (Agrawal 1999; Ali and Agrawal 2012; Xu et al. 2015). Because HAEs signal the type of damage occurring to the plant, responding appropriately to HAEs is important to mitigate further damage and begin tissue regeneration.

In natural ecosystems, plants are often attacked by multiple herbivore species, which makes defensive induction a complex task. Commonly, plants will upregulate and maintain increased specialized metabolite concentrations in response to simultaneous and sequential feedings (Kroes et al. 2016; Mertens et al. 2021; Moreira et al. 2018; Fernández De Bobadilla et al. 2021; Quijano‐Medina et al. 2023). In many cases, after the initial upregulation, they will maintain elevated defenses to prevent or reduce subsequent attacks (Gatehouse 2002). Typically defense induction occurs at the damage site, increasing defensive chemical concentrations of the affected area (Gatehouse 2002). However, defensive chemicals can be transported throughout the plant, priming areas not directly affected (i.e., systemic acquired resistance) (Kim and Felton 2013). Much of the literature surrounding defensive induction focuses on the responses to areas directly affected by herbivory, typically within leaves. However, there is a limited understanding of the cascading effects of foliar herbivory on one of the most valuable plant structures—fruits.

Fruits are rich in specialized metabolites which regulate key ecological interactions with both mutualists and antagonists (Nelson and Whitehead 2021; Cipollini and Levey 1997). Like leaves, when fruits are damaged by pests and pathogens, chemical defenses are upregulated to mitigate damaging effects and prevent future attacks. These responses are similarly mediated by the JA and SA pathways. When fruits are directly attacked, they can increase specialized metabolite concentrations to prevent further attacks (Zhou et al. 2016; Gacnik et al. 2024; Weber et al. 2021). Similarly, they can shift physiological aspects, like decreasing sugar content, weight, and pH, which makes the fruit less appealing to attackers (Wiman et al. 2015; Zhou et al. 2016; Weber et al. 2021; Casierra‐Posada et al. 2013; Peschiutta et al. 2020). Some evidence suggests plants will prime defenses in their reproductive structures when they experience foliar herbivory. Several plant species have demonstrated upregulation of defensive compounds in fruits, flowers, and seeds after foliar herbivory (McCall and Karban 2006; Baldwin and Karb 1995; Paudel et al. 2019; McArt et al. 2013). Shifts in defensive compounds can have cascading consequences on crucial interactions such as fruit removal and seed dispersal (Whitehead and Poveda 2011; McArt et al. 2013; Muñoz‐Gallego et al. 2026; Koski et al. 2017). While past studies have provided evidence supporting that foliar herbivory can alter fruit chemistry, we still have a limited understanding of the complex potential effects on fruit physiological traits, including how the effects may vary depending on herbivore identity or the extent to which multiple species are attacking plants simultaneously.

The objective of this study was to examine how varying herbivore identities and combinations during foliar herbivory shape fruit quality and specialized chemistry. To investigate this, we conducted a greenhouse experiment with ‘Albion’ strawberries (*Fragaria × ananassa* Duch). Strawberries are rich in phenolics, a class of specialized metabolites which possess a myriad of functions ranging from antiherbivory and antioxidant properties to fortifying cell walls and repairing DNA damage (Amil‐Ruiz et al. 2011; War et al. 2012; Robards et al. 1999; Haminiuk et al. 2012). We imposed damage from three species of generalist chewing lepidopteran leaf herbivores, individually and in combination, to examine how foliar herbivory altered strawberry fruit chemistry and quality. We hypothesized that: (1) foliar herbivory induces complex changes in fruit traits, including quality (sugar, weight, and pH) and specialized metabolites; (2) these changes may vary depending on the species of foliar herbivore; and (3) attack from multiple herbivores leads to more intense and complex changes, including higher diversity of specialized metabolites.

## 2 Materials and Methods

### 2.1 Study System

Strawberries (*Fragaria × ananassa* Duch) are an economically important fruit crop grown globally (Yeh et al. 2023). Strawberry fruits possess a diverse array of phenolic compounds, including anthocyanins (which compose the majority of phenolic compounds found in the fruit), flavanols, ellagitannins, proanthocyanidins, and cinnamic acid derivatives (Ornelas‐Paz et al. 2013; Buendía et al. 2010; Aaby et al. 2012). Phenolic compounds in strawberries are important for protection against abiotic and biotic stress, and contribute greatly to sensory attributes (Soto‐Vaca et al. 2012). Previous studies have shown that mechanical damage to strawberry leaves and herbivore damage to fruits can alter fruit sugar content and phenolic composition (Casierra‐Posada et al. 2013; Weber et al. 2021), and some evidence points towards increased total phenolic content in response to foliar infestation (Weber et al. 2021; Golan et al. 2017, 2015). However, little is known about the extent to which these responses may be species‐specific or how plants may respond to multiple herbivores Strawberries host a wide range of pests, including *Spodoptera frugiperda* (fall armyworm), *Heliothis virescens* (tobacco budworm), and *Helicoverpa zea* (corn earworm). These three species are generalist lepidopteran herbivores that commonly feed on a wide range of agricultural and wild host plants (Liburd and Rhodes 2019; Sheck and Gould 1996; EFSA PLH Panel 2020). All three species feed by chewing and leaving holes in plant tissue, with *H. zea* preferring to feed on fruits and flowers (EFSA PLH Panel 2020).

### 2.2 Study Materials

For plant materials, bare‐root ‘Albion’ strawberry plants were purchased from Hand Picked Nursery (Benson, North Carolina) and delivered to Virginia Polytechnic Institute and State University (Virginia Tech) on 15 January 2025. Plants were immediately transplanted to 15.2 cm diameter pots with Sun Gro Sunshine #1/Fafard 1P Mix with RESILIENCE soil (Agawam, MA) and fertilized with Osmocote Smart‐Release Plant Food Plus Outdoor and Indoor Plant Nutrient (Marysville, OH). After potting, plants were moved to the Biological Sciences Plant Growth Facility (Virginia Tech, USA, 37° 13′12″ N 80° 25′ 54″W). Plants were grown with daytime temperatures between 20°C and 24°C and nighttime temperatures between 10°C and 12°C, with ambient photoperiod. Plants were watered when soil began to dry. At anthesis, flowers were artificially self‐pollinated daily by brushing up and down the stem of the flower with an electric toothbrush. All mature flowers were artificially pollinated each day until fruit set. The first inflorescences occurred 12 February and lasted until 05 March 2025. For insect materials, *Spodoptera frugiperda* (fall armyworm), *Heliothis virescens* (tobacco budworm), and *Helicoverpa zea* (corn earworm) eggs were purchased from Benzon Research (Carlisle, PA). Insects were reared in a growth chamber with a daytime temperature of 27°C and nighttime temperature of 22°C and fed a general lepidoptera diet (Frontier Agricultural Sciences, Newark DE).

### 2.3 Herbivory Treatments and Experimental Design

Plants (*n* = 128) were arranged into 16 blocks of eight plants each, with treatments randomly arranged within each block. We had a total of eight herbivore damage treatments with different combinations of the three insect species ranging from a species richness level of zero (control) to three (Table 1). Treatments began when the first fruit began turning red (> 25% red), starting on 05 March and lasting through 21 March. To keep insects constrained to leaves, we constructed clip cages following Lemoine (2014). In short, 5 cm inner diameter pipe insulation was cut into 2.5 cm sections and mosquito netting was glued to one side of the cut pipe insulation. These cages were then placed on the upper and lower side of the leaf with insects inside and secured using staples. Three mature leaves were chosen for trap placement. All traps were placed on the terminal leaflet of the trifoliate leaf. Before treatment, the number of leaves, stems, fruits, and flowers were recorded. During treatments, 3rd‐5th instar larvae were allowed to feed in the trap for 72 h. Traps were inspected daily to ensure that feeding was occurring. In the case of insect death or if no damage had occurred after 24 h, herbivores were replaced with a new individual of the same species. After feeding, the percent herbivore damage (proportion of leaf area removed within the inner 5 cm diameter of the clip cage; Figure S1) to each leaf was assessed visually using standardized protocols (Bruna et al. 2024), always by the same individual to assure consistency.

**TABLE 1:** Experimental design of herbivore treatments.

Treatment number	Species	Species richness	Plant replicates
1	*Spodoptera frugiperda* (Sf)	1	13
2	*Heliothis virescens* (Hv)	1	14
3	*Helicoverpa zea* (Hz)	1	14
4	*Spodoptera frugiperda* + *Heliothis virescens*	2	12
5	*Spodoptera frugiperda* + *Helicoverpa zea*	2	15
6	*Heliothis virescens* + *Helicoverpa zea*	2	11
7	*Spodoptera frugiperda* + *Heliothis virescens* + *Helicoverpa zea*	3	16
Control	None	0	16

*Note:* Treatments 1–3 consisted of single‐species treatments: *Spodoptera frugiperda* (Sf), *Heliothis virescens* (Hv), and *Helicoverpa zea* (Hz), each with a species richness of 1. Treatments 4–6 included all possible two‐species combinations, and Treatment 7 included all three species simultaneously. The control treatment contained no herbivores.

### 2.4 Fruit Harvest and Tissue Sampling

Fruits were harvested 72 h after the end of treatment (144 h after insect placement). We harvested one to three fully ripe fruits per plant (depending on availability). For phenolic analysis, we collected 2 cm^3^ sections of each harvested fruit to create a composite sample per plant. Tissue samples were immediately flash frozen in liquid nitrogen and kept frozen at −80°C prior to phenolic extraction. First fruit harvest began 11 March and lasted through 27 March.

### 2.5 Fruit Quality

Fruit weight, soluble sugar content (SSC), and pH were measured for each fruit. Fruit weight (g) was recorded by weighing individual fruits as soon as they were harvested. Fruits were then juiced using a garlic press and soluble sugar content was measured as degrees Brix (°Bx) using a Vee Gee BX‐20 refractometer (Vernon Hills, IL, USA). Lastly, pH was measured using an Oakton Ecotestr PH1 Pocket pH meter (Charleston, SC, USA). Fruit quality traits were averaged per plant.

### 2.6 Phenolic Analysis

Tissue samples were lyophilized and ground into a powder and aliquots (20 mg) were taken for phenolic extractions. Before extraction, 10 μL of 200 ppm trans‐cinnamic acid was added as an internal standard to each ground sample. Extraction solvent (400 μL) of 70% methanol with 2% formic acid was added to each sample. Samples were then sonicated for 30 min and centrifuged (4°C, 10,000 rcf, 10 min). The supernatant was removed and added to a separate tube. Two additional extractions were performed on the same material, and the supernatants were combined and evaporated to dryness using a vacuum concentrator (heater: off, run time: 2 h, vacuum level: 10 Torr, ramp: 5 Torr/min). The dry samples were then stored at −80°C until HPLC analysis.

Analysis of phenolic content was carried out using an Agilent 1260 high‐performance liquid chromatograph (HPLC) equipped with a diode array detector and a Phenomenex Prodigy column (5 μm ODS‐3, 100A, 250 × 4.6 mm, model 00G‐4097‐E0). Each sample was resuspended in 100 μL of extraction solvent, and 5 μL was injected into the column for analysis. Two solvents were used for separation over a gradient: Solvent A (2% acetic acid in nanopure water) and Solvent B (100% acetonitrile). The gradient was as follows: 100% A + 0% B (3 min), 96% A + 4% B (15 min), 90% A + 10% B (30 min), 85% A + 15% B (50 min), 77% A + 23% B (60 min), 75% A + 25% B (66 min), 70% A + 30% B (70 min), 50% A + 50% B (80 min), 20% A + 80% B (83 min), 100% A + 0% B (85 min). Peaks were monitored at 280, 320, 365, and 525 nm. Post run time for each analysis was 10 min, with a 2 min delay between each new sample run.

We quantified 20 phenolic metabolites using authentic standards: catechin, epicatechin, procyanidin B1, procyanidin B2, phloridzin, syringic acid, chlorogenic acid, gentisic acid, caffeic acid, p‐coumaric acid, ferulic acid, quercetin, hyperin, isoquercitrin, quercitrin, rutin, reynoutrin, avicularin, cyanidin galactoside, and phloretin (obtained from Extrasynthese, Genay Cedex, France; Sigma‐Aldrich, St. Louis, MO, USA; and AApin Chemicals, Abingdon, Oxon, UK). A six‐point calibration curve was created using concentrations from 5 to 200 ppm of standard phenolic compounds. Peaks in samples were identified by comparison of retention time and UV spectra with authentic standards and quantified based on the calibration curves.

An additional 68 unknown compounds were classified as hydroxycinnamic acids, benzoic acids, flavonols, flavan‐3‐ols, dihydrochalcones, or anthocyanins based on UV spectra values (Table S1). Unknown compounds were semi‐quantified using the calibration curve of a known standard of the same compound class. We removed gallic acid, syringic acid, coumaric acid, and seven unknown compounds from the phenolic data set because they occurred in less than 1% of samples. Individual compound concentrations were calculated as the proportion of starting dry weight of plant material (PDW). For analyses, we summarized the total phenolic content and phenolic content per compound subclass (anthocyanins, flavonols, flavan‐3‐ols, benzoic acids, dihydrochalcones, and hydroxycinnamic acids) as the sum of individual compound concentrations. Total phenolic richness and phenolic richness per compound subclass (anthocyanins, flavonols, flavan‐3‐ols, benzoic acids, dihydrochalcones, and hydroxycinnamic acids) are the number of unique compounds detected per sample.

### 2.7 Statistical Methods

All statistical analyses were conducted in R version 4.5.1 (Posit Team 2025). To assess the effects of herbivore damage, herbivore identity, and herbivore richness on fruit quality, total phenolic content, and phenolic richness, we fit generalized linear mixed‐effects models (GLMMs) to the data using the R package glmmTMB (Brooks et al. 2017). Separate models were constructed for different response variables: average herbivory (% damage), soluble sugar content (°Bx), pH, fruit weight (g), total phenolics (PDW), and phenolic richness (number of unique compounds) (Table S2). In the average herbivory model, treatment was included as a fixed effect. In fruit quality (sugar, pH, and weight) and phenolics models, treatment was included as a fixed effect, and average herbivory (mean % damage across three treated leaves) was included as a covariate. Block ID was included as a random effect in all models to account for spatial effects. Model performance was evaluated using the check_model function in the R package performance (Lüdecke et al. 2021). Distributions were chosen to match the response variable (Table S2). To assess our specific hypotheses, we utilized planned contrasts derived from the fitted model using the R package emmeans (Lenth et al. 2018). We used three contrast sets: to assess overall effects of leaf herbivory on fruit traits (H1), we compared the undamaged control to all herbivory treatments. To assess effects of herbivore identity on fruit traits (H2), we compared single‐species treatments and the control. To assess the effect of herbivore species richness (H3), we compared one‐, two‐, and three‐species mixtures and the control. Planned contrasts were evaluated from model‐based estimated marginal means with Šidák‐adjusted *p*‐values to account for multiple comparisons, and estimates are reported as response‐scale means with associated standard errors.

## 3 Results

### 3.1 Summary of Feeding Damage Across Herbivory Treatments

Our treatments resulted in over half of the leaf area removed from each of the three leaflets that received damage per plant. Averaged across the three leaflets, herbivory over the 72‐h treatment period ranged from 66% to 71% among single‐species herbivory treatments ( *S. frugiperda* : 66.2% ± 6.9%, *H. virescens* : 71.0% ± 7.1%, *H. zea* : 48.6% ± 7.6%). Post hoc contrasts indicated strong differences between some species treatments (Sf vs. Hz, *p* = 0.025; Hv vs. Hz: *p* = 0.002; Table S3). Average herbivory ranged from 58% to 66% among treatment groups that varied in herbivore species richness (single species: 61.9% ± 4.3%, two species: 58.2% ± 4.0%, three species: 65.8% ± 7.5%). Post hoc contrasts indicated no supported differences between richness levels (Table S3).

### 3.2 (H1) Does Foliar Herbivory Induce Changes in Fruit Quality and Specialized Metabolites?

#### 3.2.1 Fruit Quality

There were no supported differences between undamaged and damaged plants in soluble sugar content, average fruit weight, or total fruit pH (Table S4, Figure S2). The overall model for fruit pH indicated a strong effect of average herbivory (Table S2, Figure S3). Model estimates showed a decrease from 3.59 (±0.06) pH at 0% herbivory to 3.35 (±0.07) pH at 100% herbivory.

#### 3.2.2 Phenolic Content

There were no supported differences between undamaged and damaged treatments in phenolic content and content by phenolic subclass: anthocyanins, flavan‐3‐ols, flavonols, benzoic acids, dihydrochalcones, and hydroxycinnamic acids (Table S5, Figure S4).

#### 3.2.3 Phenolic Richness

**FIGURE 1:**
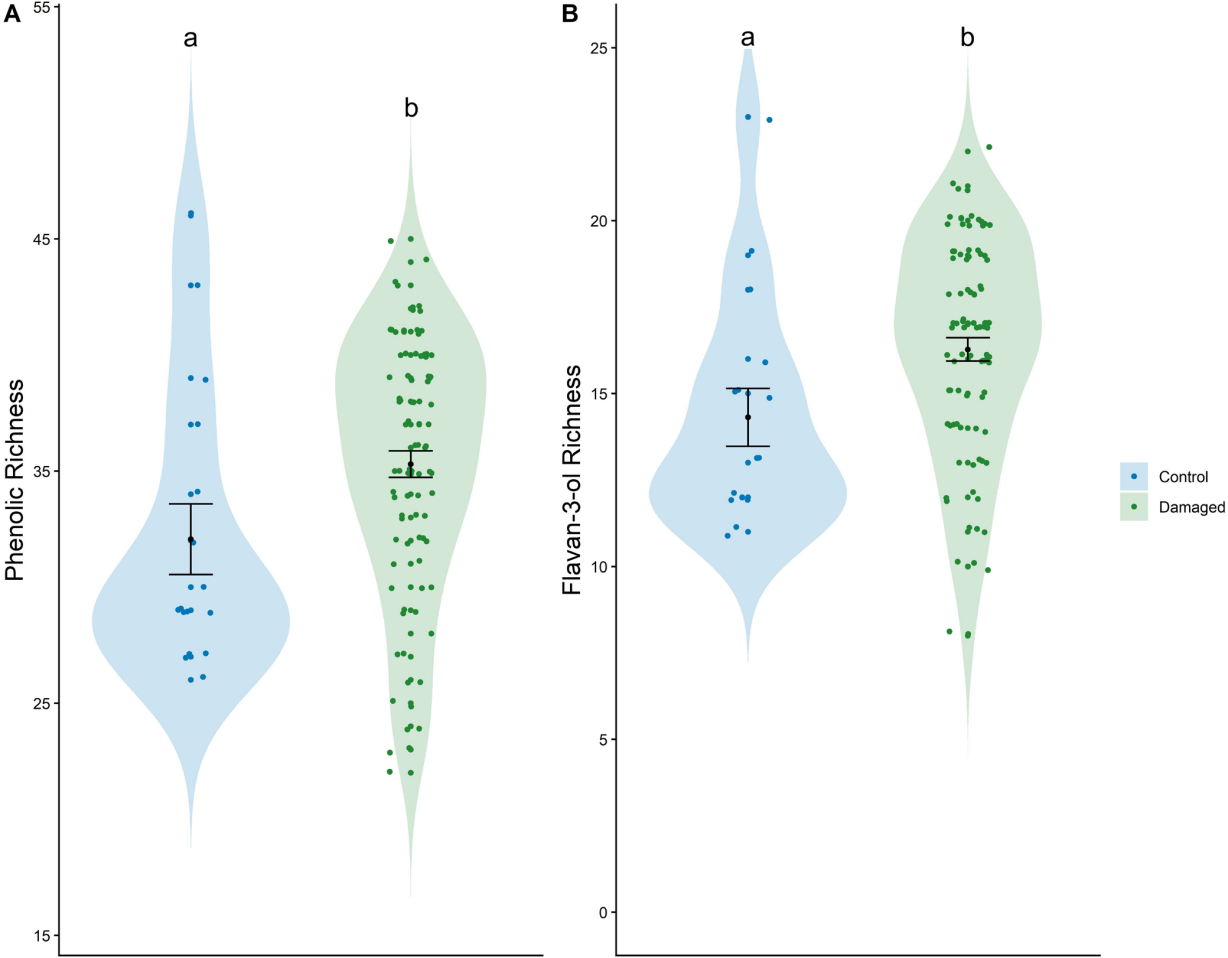
Effects of foliar damage on strawberry fruit phenolic richness (A) and flavan‐3‐ol richness (B). Colored points in all figures represent individual strawberry plants (*N* = 106). Black points and error bars indicate the mean ± standard error. Letters above plots indicate Šidák post hoc contrasts among treatments supported at *p* < 0.05 (Table S6).

There were supported differences in phenolic richness between damaged and undamaged plants (Table S6; Figure 1). Phenolic richness was 13% higher in damaged plants relative to undamaged controls (*p* = 0.0197). By compound class, flavan‐3‐ol richness was 15% higher in damaged plants (*p* = 0.0429), while dihydrochalcone richness was 34% higher in damaged plants, though with weaker statistical support (*p* = 0.0787) (Table S6; Figure 1). There were no supported differences among the remaining compound classes (Table S6, Figure S5).

### 3.3 (H2) do the Effects of Herbivory Vary Depending on Herbivore Identity?

#### 3.3.1 Fruit Quality

**FIGURE 2:**
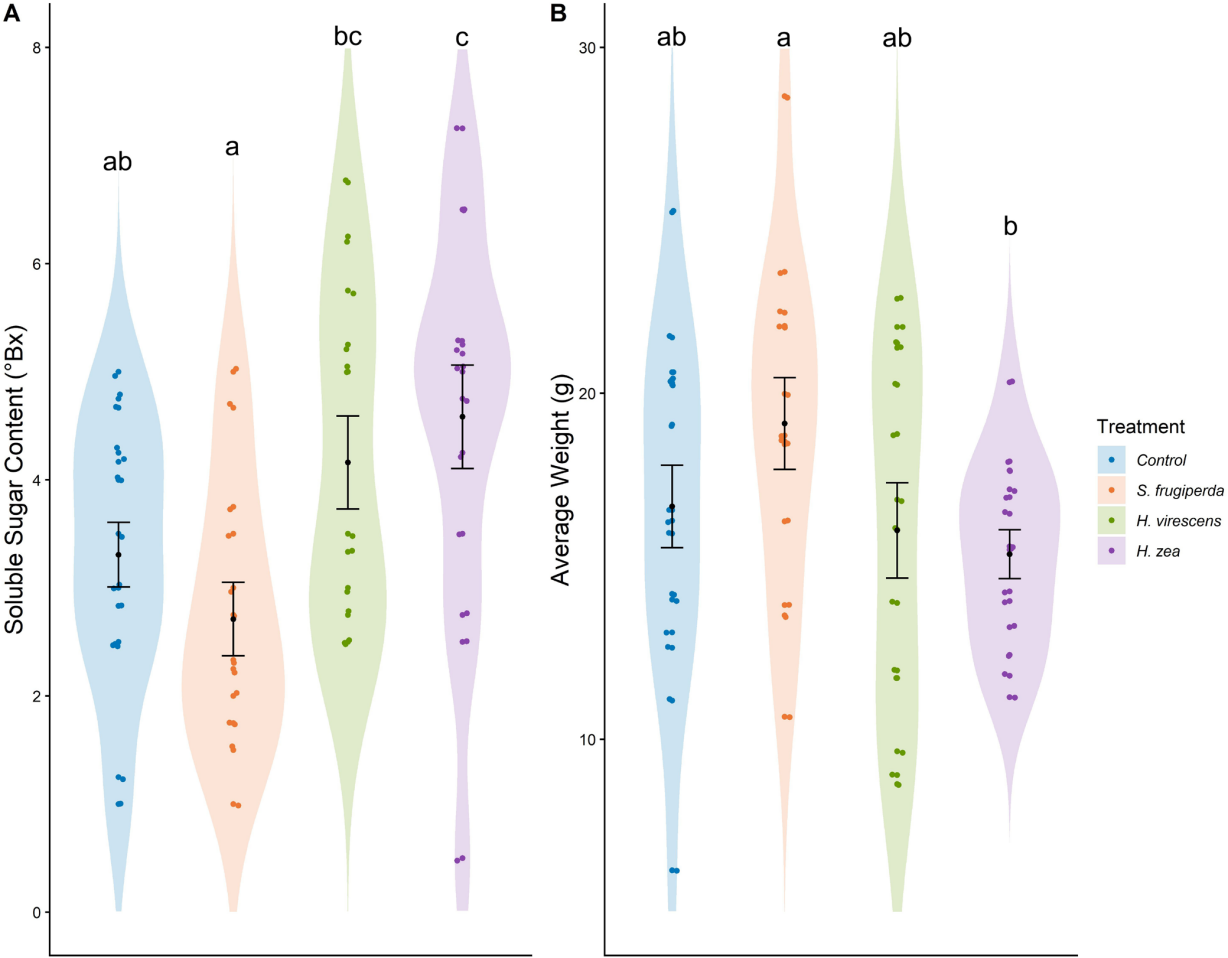
Effects of foliar damage from three different herbivores on strawberry fruit soluble sugar content (A) and average weight (B). Colored points in all figures represent individual strawberry plants (*N* = 57). Black points and error bars indicate the mean ± standard error. Letters above plots indicate Šidák post hoc contrasts among treatments supported at *p* < 0.05 (Table S4).

For soluble sugar content, post hoc contrasts supported differences among single‐species treatments (Table S4; Figure 2). Soluble sugar content was 42% higher in plants damaged by *H. virescens* (*p* = 0.008) and 52% higher in plants damaged by *H. zea* than *S. frugiperda* (*p* = 0.0007). Relative to the control, soluble sugar content was 32% higher in plants damaged by *H. zea* (*p* = 0.0333). For average weight, there were supported differences among single‐species treatments (Table S4; Figure 2). Average weight was 20% lower in plants treated with *H. zea* than *S. frugiperda* (*p* = 0.0479) and 19% lower in plants treated with *H. virescens* than *S. frugiperda* (*p* = 0.0558). There were no supported differences in fruit pH among single‐species treatments and controls (Table S4, Figure S6).

#### 3.3.2 Phenolic Content

**FIGURE 3:**
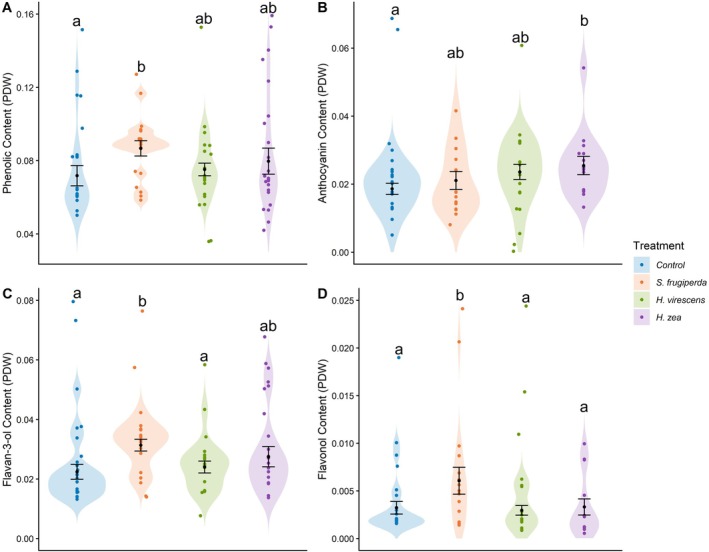
Effects of foliar damage from three different herbivores on strawberry fruit phenolic content (A), anthocyanin content (B), flavan‐3‐ol content (C), and flavonol content (D). Colored points in all figures represent individual strawberry plants (*N* = 57). Black points and error bars indicate the mean ± standard error. Letters above plots indicate significant contrasts (*p* < 0.05) between treatments based on Šidák post hoc analyses (Table S5).

For total phenolic content, post hoc contrasts did not support differences among single‐species treatments, but did support differences between plants treated with *S. frugiperda* and the control (Table S5; Figure 3). Plants damaged by *S. frugiperda* showed 6% higher total phenolic content than controls (*p* = 0.035). These effects varied substantially across compound sub‐class. For anthocyanins, there were no supported differences among single‐species treatments, but plants damaged by *H. zea* had 22% higher anthocyanin content than controls (*p* = 0.0454). For flavan‐3‐ols, plants damaged by *S. frugiperda* had 27% higher content than plants damaged by *H. virescens* (*p* = 0.0357) and 34% higher content than controls (*p* = 0.0284) (Table S5; Figure 3). For flavonols, plants treated with *S. frugiperda* had 56% higher content than plants treated with *H. virescens* (*p* = 0.0126) and 57% higher than plants treated with *H. zea* (*p* = 0.0089) (Table S5; Figure 3). For benzoic acids, plants treated with *S. frugiperda* were 31% higher in benzoic acid content than *H. zea* (*p* = 0.0545) and 33% higher than the control (*p* = 0.0745) (Table S5; Figure 3). Lastly, for dihydrochalcones and hydroxycinnamic acids, there were no supported contrasts across treatments (Table S5; Figure S7).

#### 3.3.3 Phenolic Richness

**FIGURE 4:**
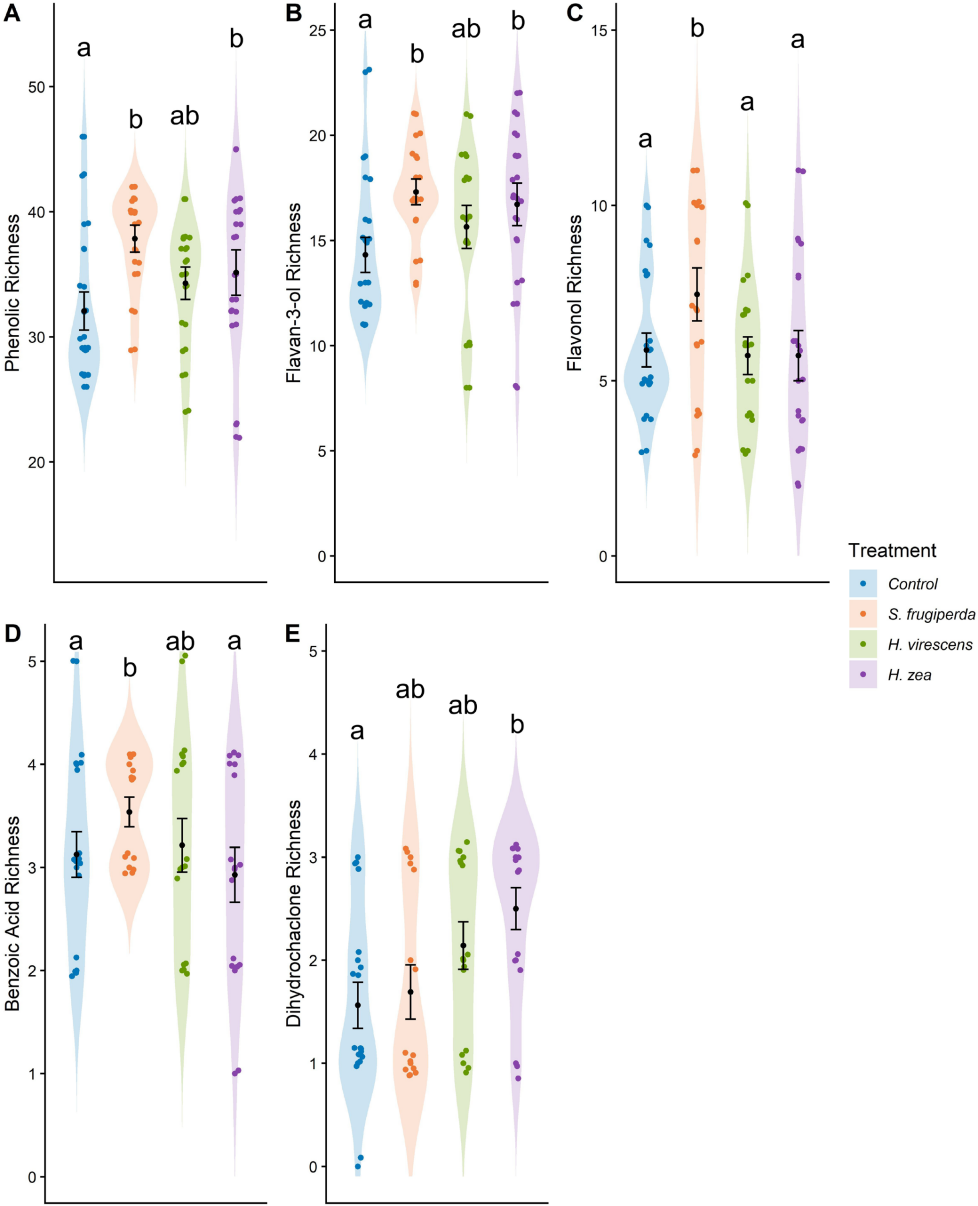
Effects of foliar damage from three different herbivores on strawberry fruit phenolic richness (A), flavan‐3‐ol richness (B), flavonol richness (C), benzoic acid richness (D), and dihydrochalcone richness (E). Colored points in all figures represent individual strawberry plants (*N* = 57). Black points and error bars indicate the mean ± standard error. Letters above plots indicate significant contrasts (*p* < 0.05) between treatments based on Šidák post hoc analyses (Table S6).

For phenolic richness, post hoc analyses did not support differences among single‐species treatments; however, they did support differences between treatments and the control (Table S6; Figure 4). Compared to the control, overall phenolic richness was 13% higher in plants damaged by *H. zea* (*p* = 0.0395) and 21% higher in plants damaged by *S. frugiperda* (*p* = 0.0045). These effects varied substantially across compound sub‐class (Table S6; Figure 4). For flavan‐3‐ol richness, there were supported differences between single‐species treatments and the controls, with no differences among single‐species treatments alone (Table S6; Figure 4). Compared to the control, plants damaged by *S. frugiperda* had 22% higher flavan‐3‐ol richness (*p* = 0.0195) and plants damaged by *H. zea* had 18% higher flavan‐3‐ol richness (*p* = 0.0389). For flavonol richness, there were supported differences among single‐species treatments and compared to the control (Table S6; Figure 4). Plants damaged by *S. frugiperda* had 25% higher flavonol richness than *H. virescens* (*p* = 0.0494) and 28% higher than *H. zea* (*p* = 0.0355). Additionally, flavonol richness was 34% higher in plants damaged by *S. frugiperda* than the control (S1 vs. C: −2.211 ± 1.05, *p* = 0.0371). For benzoic acids, there were supported differences among single‐species treatments and compared to the control (Table S6; Figure 4). Benzoic acid richness was 22% higher in plants damaged by *S. frugiperda* than *H. zea* (*p* = 0.0304) and 25% higher in *S. frugiperda* than the control (*p* = 0.0416). For dihydrochalcone richness, there were supported differences among single‐species treatments and compared to the control (Table S6; Figure 4). Plants damaged by *H. zea* showed 36% higher richness than *S. frugiperda* (*p* = 0.023) and 41% higher in dihydrochalcone richness than the control (*p* = 0.0041). Additionally, plants damaged by *H. virescens* had 41% higher dihydrochalcone richness than the control (*p* = 0.0683). Lastly, there were no supported differences in anthocyanin richness and hydroxycinammic acid richness (Table S6; Figure S8).

### 3.4 (H3) Does Attack From Multiple Herbivores Lead to More Intense and Complex Changes in Fruit Traits?

#### 3.4.1 Fruit Quality

**FIGURE 5:**
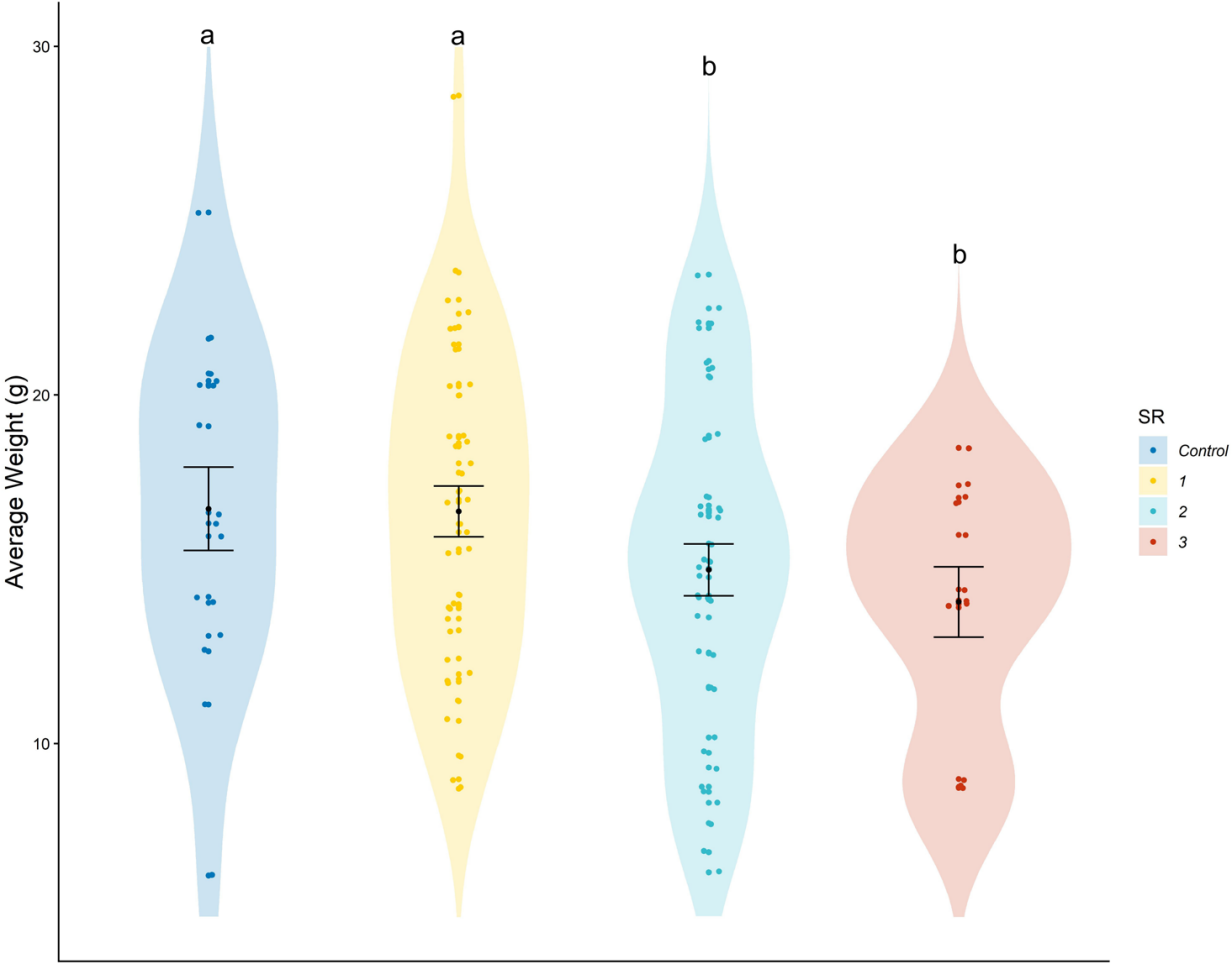
Effects of foliar damage from three levels of herbivore species richness on strawberry fruit average weight. Colored points represent individual strawberry plants (*N* = 106). Black points and error bars indicate the mean ± standard error. Letters above plot indicate significant contrasts (*p* < 0.05) between treatments based on Šidák post hoc analyses (Table S4).

There were supported differences in average fruit weight among species richness levels and compared to the control (Table S4; Figure 5). Average weight saw a directional decrease; compared to a species richness of one, average weight was 10% lower at a species richness of two (*p* = 0.0467) and 18% lower at a species richness level of three (*p* = 0.0445). Compared to the control, average weight was 21% lower at a species richness of two (*p* = 0.0524) and 28% lower at a species richness of three (*p* = 0.0421). There were no supported differences in soluble sugar content and total pH (Table S4, Figure S9).

#### 3.4.2 Total Phenolic Content

There were no supported differences in total phenolics or phenolic content by subclass (Table S5, Figure S10).

#### 3.4.3 Phenolic Richness

**FIGURE 6:**
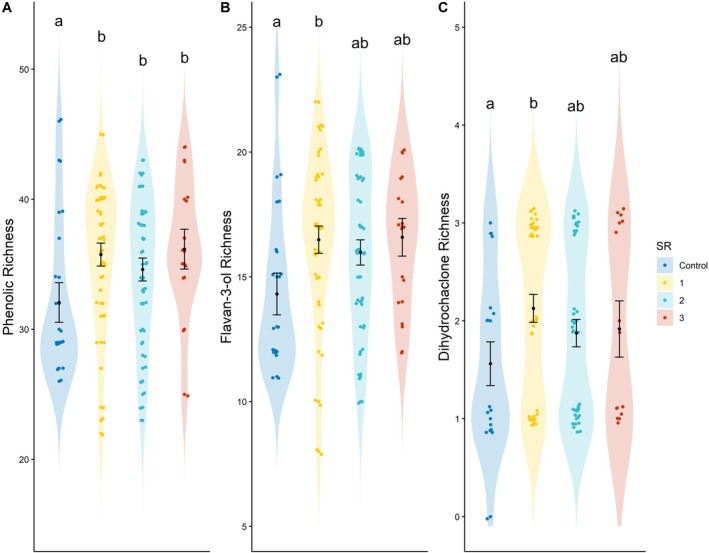
Effects of foliar damage from three levels of herbivore species richness on strawberry fruit phenolic richness (A), flavan‐3‐ol richness (B), and dihydrochalcone richness (C). Colored points in all figures represent individual strawberry plants (*N* = 106). Black points and error bars indicate the mean ± standard error. Letters above plots indicate significant contrasts (*p* < 0.05) between treatments based on Šidák post hoc analyses (Table S6).

For phenolic richness, post hoc analyses supported differences between species richness treatments and the control (Table S6; Figure 6). Phenolic richness was 15% higher at a richness level of one (*p* = 0.0134), 11% higher at a richness level of two (*p* = 0.0547), and 16% higher at a richness level of three (*p* = 0.0268). These effects varied substantially across compound sub‐class (Table S6; Figure 6). For flavan‐3‐ol richness, there were supported differences between species richness treatments and the controls, with no differences among species richness treatments alone (Table S6; Figure 6). Flavan‐3‐ol richness was 18% higher at a richness level of one (*p* = 0.0318) and 18% higher at a richness level of three (*p* = 0.0663) when compared to the control. For dihydrochalcone richness, there were supported differences between species richness treatments and the controls, with no differences among species richness treatments alone (Table S6; Figure 6). Dihydrochalcone richness was 42% higher at a richness level of one (*p* = 0.0423) compared to the control. Lastly, there were no supported contrasts with anthocyanin richness, flavonol richness, benzoic acid richness, and hydroxycinamic acid richness (Table S6, Figure S11).

## 4 Discussion

Exploring how foliar herbivory influences fruit quality and specialized metabolism is critical for understanding both the mechanisms and ecological consequences of induced plant defenses. Through a greenhouse experiment with strawberries, we examined how the presence of foliar herbivory, herbivore identity, and herbivore species richness affected fruit sugar content, weight, pH, phenolic content, and phenolic richness. We investigated three hypotheses and found that: (1) foliar herbivory generally increased fruit phenolic richness and lowered pH, (2) the direction and magnitude of quality and chemical responses depended on herbivore identity, and (3) higher herbivore species richness led to decreased fruit weight. Together, these results indicate that foliar herbivory is an important factor shaping fruit traits, with outcomes shaped by the identity and diversity of herbivores involved. Our findings point to species‐specific elicitation mechanisms during foliar herbivory, even among closely related generalist herbivores.

### 4.1 Foliar Damage Decreases pH and Increases Phenolic Richness

Foliar herbivory had pronounced effects on several aspects of fruit chemistry and quality. In particular, across all herbivory treatments, we observed a consistent increase in phenolic richness. We did observe a slight increase in total phenolic content in response to foliar herbivory, however this trend was not statistically supported. This could indicate that we failed to capture some aspect or waited too little or too long after feeding to process fruits. Previous research into the effects of foliar herbivory on fruit chemistry have demonstrated increases in total phenolic content (McCall and Karban 2006; Baldwin and Karb 1995; Paudel et al. 2019). Changes in total phenolic content in strawberry leaves has been demonstrated to be highly variable, with documented increases occurring between 1 h and 1 week after feeding (Golan et al. 2015).

The increase in phenolic richness is consistent with prior knowledge demonstrating that foliar herbivory can induce greater phenolic compound diversity (Wallis and Galarneau 2020). In particular, we detected increased flavonol and flavan‐3‐ol richness in fruits from damaged plants. These phenolic subclasses are commonly synthesized in response to herbivore attack and are well known components of plant defensive chemistry (Simmonds 2003). In addition to effects on phenolics, we found some evidence that foliar herbivory can decrease fruit pH. Although there was no statistical support for an overall difference in pH between control and herbivore‐damaged plants, we saw a negative relationship between average herbivory (% tissue removed) and fruit pH. A negative relationship between foliar herbivory and fruit pH has been documented previously in strawberries, where juice pH declined in proportion to the extent of defoliation (Casierra‐Posada et al. 2013). Similar patterns have been reported in cherry fruits, which exhibit increased acidity during sawfly infestations (Peschiutta et al. 2020). Hebrivory can cause an increase in the production of phenolic acids and reactive oxygenated species (ROS), leading to a decrease in pH throughout various plant structures (Bi and Felton 1995). It's important to note that we conducted this experiment with a domesticated plant; domestication reduces plant defenses due to selective breeding and stress control which do not reflect natural conditions (Whitehead and Poveda 2019; Whitehead et al. 2017).

### 4.2 Different Herbivore Species Elicited Different Chemical and Quality Responses

All three species of herbivores in this study were generalist chewing feeders in the same family (Noctuidae), yet we still observed different responses across the measured traits. There is strong past evidence for distinct plant responses to different feeding guilds; notably between chewing and puncture feeders (Agrawal 1999; Fernández De Bobadilla et al. 2022; Mertens et al. 2021; Xiao et al. 2019). Similarly, there is general understanding that plants will respond differently to generalist and specialist species, even if they belong to the same family (Ali and Agrawal 2012; Van Zandt and Agrawal 2004). Less is known about mechanisms driving specificity of plant responses to different generalist herbivores in the same family. Differences in plant responses to different herbivores in this study could be explained by the average proportion of herbivory between the single‐species treatments, though the effects of average herbivory were accounted for in our models and were overall minimal. Alternatively, species‐specific responses could have been driven by differences in the composition or concentration of herbivore‐associated elicitors (HAEs) in the saliva and frass of the insects. Most lepidopteran larvae produce an HAE known as volicitin (N‐linolenoyl‐L‐glutamine), which has been shown to induce defensive responses in many different species of plants (Bonaventure et al. 2011). In the case of our species, *H. zea* and *H. virescens* saliva and frass have been shown to contain different amounts of volicitin, suggesting that they can elicit different defensive responses on the same plant (Mori et al. 2001). Unique responses to *S. frugiperda* could be explained by specialized adaptations this species has to delay induced responses during herbivory (De Lange et al. 2020). It has also been suggested that *S. frugiperda* gut microbiota produce enzymes that are responsible for downregulating plant defensive responses, allowing for more consumption of plant material (Acevedo et al. 2017; Chuang et al. 2014).

Although deciphering the specific elicitors involved would require further study, we did document distinct responses of fruits to different species of foliar herbivores. Damage from *S. frugiperda* was associated with lower soluble sugar content and higher fresh weight, while damage from *H. virescens* and *H. zea* was associated with higher soluble sugar content and lower fresh weight. The inverse relationship between soluble sugar content and average fruit weight is consistent with our knowledge that fruit water content dilutes soluble sugars in the fruit. However, the mechanism driving differences in weight and sugar based on insect herbivore identity is unclear. Foliar herbivory can have cascading effects of reduced photosynthesis and water content in undamaged tissue (Zangerl et al. 2002; Nabity et al. 2009), however, this may not be consistent in reproductive tissues, and we would expect a consistent response, regardless of herbivore species identity. Decreases in soluble sugar content following herbivory or leaf removal have been documented in multiple systems, including cherries and strawberries (Casierra‐Posada et al. 2013; Peschiutta et al. 2020). Conversely, some studies have found the opposite effect with increased sugar content after experiencing leaf damage (Ibanez et al. 2019). Some studies suggest that induction of defenses can reduce fruit count but increase fruit weight, while others have documented decreased fruit production and weight (Redman et al. 2001; Casierra‐Posada et al. 2013).

Fruit phenolic content and richness also responded differently to different herbivores. Notably fruit flavonol and flavan‐3‐ol content and richness increased most strongly in response to *S. frugiperda* herbivory, while fruit dihydrochalcone richness and anthocyanin content increased most strongly in response to *H. zea*. The broader flavonoid class which includes flavonols and dihydrochalcones is largely responsible for deterring herbivory, modulating oxidative stress, and regulating auxin transport (Treutter 2005; Buer et al. 2010; Simmonds 2003). An increase in the content and richness of these compounds is what we expected to occur, but further work is needed to understand whether the specific responses to different herbivores represent adaptive changes that optimize plant resistance to each species.

Plant defense induction can be incredibly variable from system to system. A few studies have seen similar phenolic content responses in the same plant species when attacked by different herbivores of the same feeding guild (Moreira et al. 2015; Eisenring et al. 2018). Based on our results, we believe that there is a molecular difference in defense elicitation among our three species. Although strawberry fruit maturation is primarily regulated by abscisic acid (ABA), jasmonic acid (JA) also contributes to fruit development by regulating sugar metabolism and the induction of defense‐related secondary metabolites (Garrido‐Bigotes et al. 2018; Han et al. 2019). Previous work has shown that *S. frugiperda* possesses salivary effectors capable of suppressing or delaying JA defensive pathways in some host plants (Acevedo et al. 2017; Chuang et al. 2014). If similar mechanisms occur in strawberry, partial suppression of JA signaling could explain the reduced soluble sugar content and increased fruit weight observed following *S. frugiperda* herbivory. However, this hypothesis is complicated by the increase in flavan‐3‐ol and flavonol concentration and richness. Additional work examining hormone signaling and herbivore‐associated elicitors is needed to determine how these herbivores differentially regulate strawberry defense responses.

### 4.3 Variable Effects by Increased Species Richness

We observed a directional decrease in the average fruit weight of strawberries as herbivore species richness increased. To our knowledge, this observation is previously undocumented in literature. Removal of leaf tissue has been demonstrated to decrease strawberry fruit weight (Casierra‐Posada et al. 2013). Because fruit weight and soluble sugar content are interconnected traits, there should have been a similar directional change with soluble sugar content; however, this did not occur. The mechanism behind why fruit weight decreased is unclear, and future studies should explore this phenomenon in a more controlled setting. A possible explanation could be that amplified defensive expression could inhibit growth processes through a decrease in available resources for metabolite synthesis, leading to a decrease in fruit size, but this does not explain why there was not a clear trend with sugar content.

We did not find support for our hypothesis that increased species richness during foliar herbivory would increase phenolic richness. We observed strong increases in phenolic richness compared to the control, and a semi‐directional increase across richness levels, but no supported differences among species richness levels. We also observed similar non‐supported trends among compound subclasses, including anthocyanins, flavonols, flavan‐3‐ols, benzoic acids, and dihydrochalcones. Overall, these findings suggest that foliar herbivory, regardless of species richness, increased phenolic richness. We expected to see an increase in phenolic diversity as species richness increased, in line with the interaction diversity hypothesis that posits that plants must produce a diverse set of chemicals in order to counter a diverse set of antagonists (Berenbaum et al. 1986; Whitehead et al. 2022). Our results could have been potentially skewed by some of the opposite interactions we observed among the single‐species treatments, indicating that one or more species might be inhibiting defensive induction.

Induced defenses are highly complex, and the literature reports a wide range of inconsistent findings with combinations of generalist and specialist species, as well as richness with different feeding guilds. The variability in responses seen across the literature suggests that the effects of multiple herbivores may depend on the diversity of feeding guilds, sequence of attackers, and timing of attack (Fernández De Bobadilla et al. 2021; Moreira et al. 2015; Mathur et al. 2013). Future studies should tease apart mechanistic responses of fruit chemistry to multiple herbivore attackers, including: induction peak/timing and HAEs of the insect species.

## 5 Conclusions

This study provides key insight into the complexity of plant–insect herbivore interactions by demonstrating that attack by different combinations of closely related generalist insect herbivores can produce distinct effects on fruit quality and chemical composition. Our results indicate that defensive upregulation in response to foliar herbivory can carry over into fruits, altering both their chemistry and potential nutritional value. Future studies should extend this work by examining how herbivore identity and composition of herbivores during foliar attack influence other reproductive structures, such as flowers and seeds, as well as downstream effects on ecosystem services like frugivory and seed dispersal.

## Author Contributions

**Xavier Ozowara:** conceptualization (lead), data curation (lead), formal analysis (lead), funding acquisition (equal), investigation (lead), methodology (lead), project administration (lead), writing – original draft (lead). **Taylor M. Sloey:** conceptualization (supporting), investigation (supporting), supervision (supporting), writing – review and editing (equal). **Susan R. Whitehead:** conceptualization (supporting), formal analysis (supporting), funding acquisition (equal), investigation (supporting), methodology (equal), supervision (lead), writing – review and editing (equal).

## Funding

This work was supported by the Foundation for Food and Agriculture Research (FF‐NIA19‐0000000028).

## Conflicts of Interest

The authors declare no conflicts of interest.

## Supporting information


**Figure S1:** Examples of leaf damage by herbivores within clip cages. After feeding trials, the proportion of leaf area removed within the inner 5 cm diameter of the clip cage was assessed visually to determine the percentage of leaf removal.
**Figure S2:** Effects of foliar damage on strawberry fruit average weight (A), soluble sugar content (B), and pH (C). Colored points in all figures represent individual strawberry plants (*N* = 106). Black points and error bars indicate the mean ± standard error.
**Figure S3:** Mean fruit pH across average herbivory (% tissue removed). Lines represent the estimated model fits and the gray areas surrounding the lines capture standard deviation around those estimates. Points represent individual plants (*N* = 106).
**Figure S4:** Effects of foliar damage on strawberry fruit phenolic content (A), anthocyanin content (B), flavan‐3‐ol content (C), flavonol content (D), benzoic acid content (E), hydroxycinammic acid content (F), and dihydrochalcone content (G). Colored points in all figures represent individual strawberry plants (*N* = 106). Black points and error bars indicate the mean ± standard error.
**Figure S5:** Effects of foliar damage on strawberry fruit anthocyanin richness (A), flavonol richness (B), benzoic acid richness (C), hydroxycinammic acid richness (D), and dihydrochalcone richness (E). Colored points in all figures represent individual strawberry plants (*N* = 106). Black points and error bars indicate the mean ± standard error.
**Figure S6:** Effects of foliar damage from three different herbivores on strawberry fruit pH. Colored points in all figures represent individual strawberry plants (*N* = 57). Black points and error bars indicate the mean ± standard error.
**Figure S7:** Effects of foliar damage from three different herbivores on strawberry fruit benzoic acid content (A), hydroxycinammic acid content (B), and dihydrochalcone content (C). Colored points in all figures represent individual strawberry plants (*N* = 57). Black points and error bars indicate the mean ± standard error.
**Figure S8:** Effects of foliar damage from three different herbivores on strawberry fruit anthocyanin richness (A) and hydroxycinammic acid richness (B). Colored points in all figures represent individual strawberry plants (*N* = 57). Black points and error bars indicate the mean ± standard error.
**Figure S9:** Effects of foliar damage from three levels of herbivore species richness on strawberry fruit soluble sugar content (A) and pH (B). Colored points in all figures represent individual strawberry plants (*N* = 57). Black points and error bars indicate the mean ± standard error.
**Figure S10:** Effects of foliar damage from three levels of herbivore species richness on strawberry fruit phenolic content (A), anthocyanin content (B), flavan‐3‐ol content (C), flavonol content (D), benzoic acid content (E), hydroxycinammic acid content (F), and dihydrochalcone content (G). Colored points in all figures represent individual strawberry plants (*N* = 107). Black points and error bars indicate the mean ± standard error.
**Figure S11:** Effects of foliar damage from three levels of herbivore species richness on strawberry fruit anthocyanin richness (A), flavonol richness (B), benzoic acid richness (C), and hydroxycinammic acid richness (D), and dihydrochalcone content (G). Colored points in all figures represent individual strawberry plants (*N* = 107). Black points and error bars indicate the mean ± standard error.
**Table S1:** Detected compounds, compound classes based on spectra data, and mean (± se) concentration for each treatment (Treatment 1: *Spodoptera frugiperda* ; Treatment 2: *Heliothis virescens* ; Treatment 3: *Helicoverpa zea* ; Treatment 4: *Spodoptera frugiperda* + *Heliothis virescens* ; Treatment 5: *Spodoptera frugiperda* + *Helicoverpa zea* ; Treatment 6: *Heliothis virescens* + *Helicoverpa zea* ; Treatment 7: *Spodoptera frugiperda* + *Heliothis virescens* + *Helicoverpa zea* ).
**Table S2:** Generalized linear mixed model outputs showing the effects of treatments on fruit quality (soluble sugar content, average weight, pH), phenolic content and content by subclass (anthocyanins, flavonols, flavan‐3‐ols, benzoic acids, dihydrochalcones, and hydroxycinnamic acids), and phenolic richness and richness by subclass (anthocyanins, flavonols, flavan‐3‐ols, benzoic acids, dihydrochalcones, and hydroxycinnamic acids).
**Table S3:** Planned contrasts evaluating the effect of species identity (H2) and species richness (H3) on average herbivory. Sf: *Spodoptera frugiperda* ; Hv: *Heliothis virescens* ; Hz: *Helicoverpa zea* ; SR1: Treatments 1, 2, and 3; SR2: Treatments 4, 5, and 6; SR3: Treatment 7.
**Table** S4. Planned contrasts evaluating the effect of herbivore damage (H1), species identity (H2), and species richness (H3) on fruit quality (soluble sugar content, average weight, pH). Sf: *Spodoptera frugiperda* ; Hv: *Heliothis virescens* ; Hz: *Helicoverpa zea* ; SR1: Treatments 1, 2, and 3; SR2: Treatments 4, 5, and 6; SR3: Treatment 7.
**Table S5:** Planned contrasts evaluating the effect of herbivore damage (H1), species identity (H2), and species richness (H3) on fruit quality phenolic content and content by subclass (anthocyanins, flavonols, flavan‐3‐ols, benzoic acids, dihydrochalcones, and hydroxycinnamic acids). Sf: *Spodoptera frugiperda* ; Hv: *Heliothis virescens* ; Hz: *Helicoverpa zea* ; SR1: Treatments 1, 2, and 3; SR2: Treatments 4, 5, and 6; SR3: Treatment 7.
**Table S6:** Planned contrasts evaluating the effect of herbivore damage (H1), species identity (H2), and species richness (H3) on fruit quality phenolic richness and richness by subclass (anthocyanins, flavonols, flavan‐3‐ols, benzoic acids, dihydrochalcones, and hydroxycinnamic acids). Sf: *Spodoptera frugiperda* ; Hv: *Heliothis virescens* ; Hz: *Helicoverpa zea* ; SR1: Treatments 1, 2, and 3; SR2: Treatments 4, 5, and 6; SR3: Treatment 7.

## Data Availability

Full data set and R scripts can be found in our Github repository: https://github.com/WhiteheadLabVT/‐Herbivore‐identity‐and‐species‐richness‐shape‐fruit‐chemical‐and‐quality‐responses‐to‐foliar‐herbiv.
