# Supplementary material for: Herbivore Identity and Species Richness Shape Fruit Chemical and Quality Responses to Foliar Herbivory
Source: Ecol Evol. 2026 Jul 7;16(7):e73970. doi: 10.1002/ece3.73970 (PMC13341151; doi:10.1002/ece3.73970)
Supplement: Supplementary file 1 — Figure S1: Examples of leaf damage by herbivores within clip cages. After feeding trials, the proportion of leaf area removed within the inner 5 cm diameter of the clip cage was assessed visually to determine the percentage of leaf removal. Figure S2: Effects of foliar damage on strawberry fruit average weight (A), soluble sugar content (B), and pH (C). Colored points in all figures represent individual strawberry plants (N = 106). Black points and error bars indicate the mean ± standard error. Figure S3: Mean fruit pH across average herbivory (% tissue removed). Lines represent the estimated model fits and the gray areas surrounding the lines capture standard deviation around those estimates. Points represent individual plants (N = 106). Figure S4: Effects of foliar damage on strawberry fruit phenolic content (A), anthocyanin content (B), flavan‐3‐ol content (C), flavonol content (D), benzoic acid content (E), hydroxycinammic acid content (F), and dihydrochalcone content (G). Colored points in all figures represent individual strawberry plants (N = 106). Black points and error bars indicate the mean ± standard error. Figure S5: Effects of foliar damage on strawberry fruit anthocyanin richness (A), flavonol richness (B), benzoic acid richness (C), hydroxycinammic acid richness (D), and dihydrochalcone richness (E). Colored points in all figures represent individual strawberry plants (N = 106). Black points and error bars indicate the mean ± standard error. Figure S6: Effects of foliar damage from three different herbivores on strawberry fruit pH. Colored points in all figures represent individual strawberry plants (N = 57). Black points and error bars indicate the mean ± standard error. Figure S7: Effects of foliar damage from three different herbivores on strawberry fruit benzoic acid content (A), hydroxycinammic acid content (B), and dihydrochalcone content (C). Colored points in all figures represent individual strawberry plants (N = 57). Black points and error b [file ECE3-16-e73970-s001.docx]

**Supplementary Materials**

# **Figures**


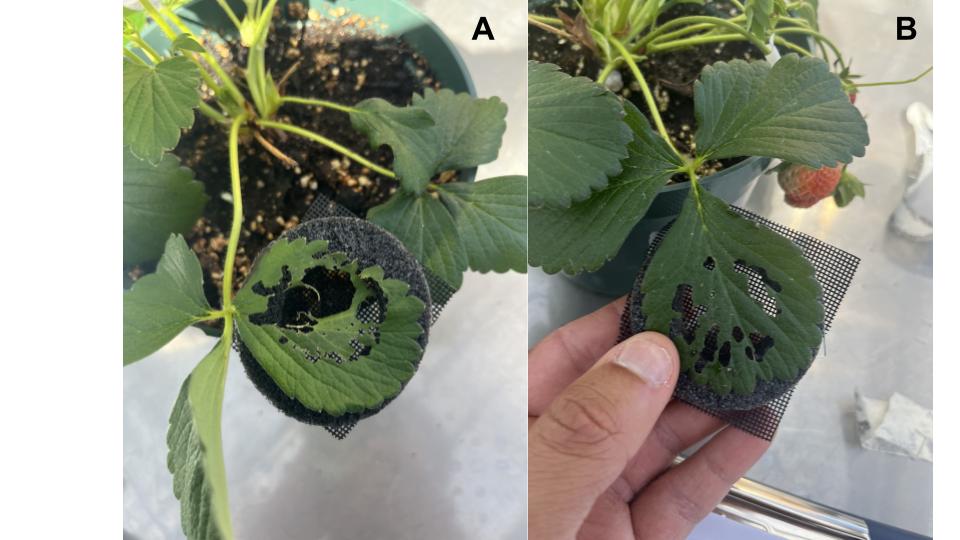


**Figure S1**. Examples of leaf damage by herbivores within clip cages. After feeding trials, the proportion of leaf area removed within the inner 5 cm diameter of the clip cage was assessed visually to determine the percentage of leaf removal.


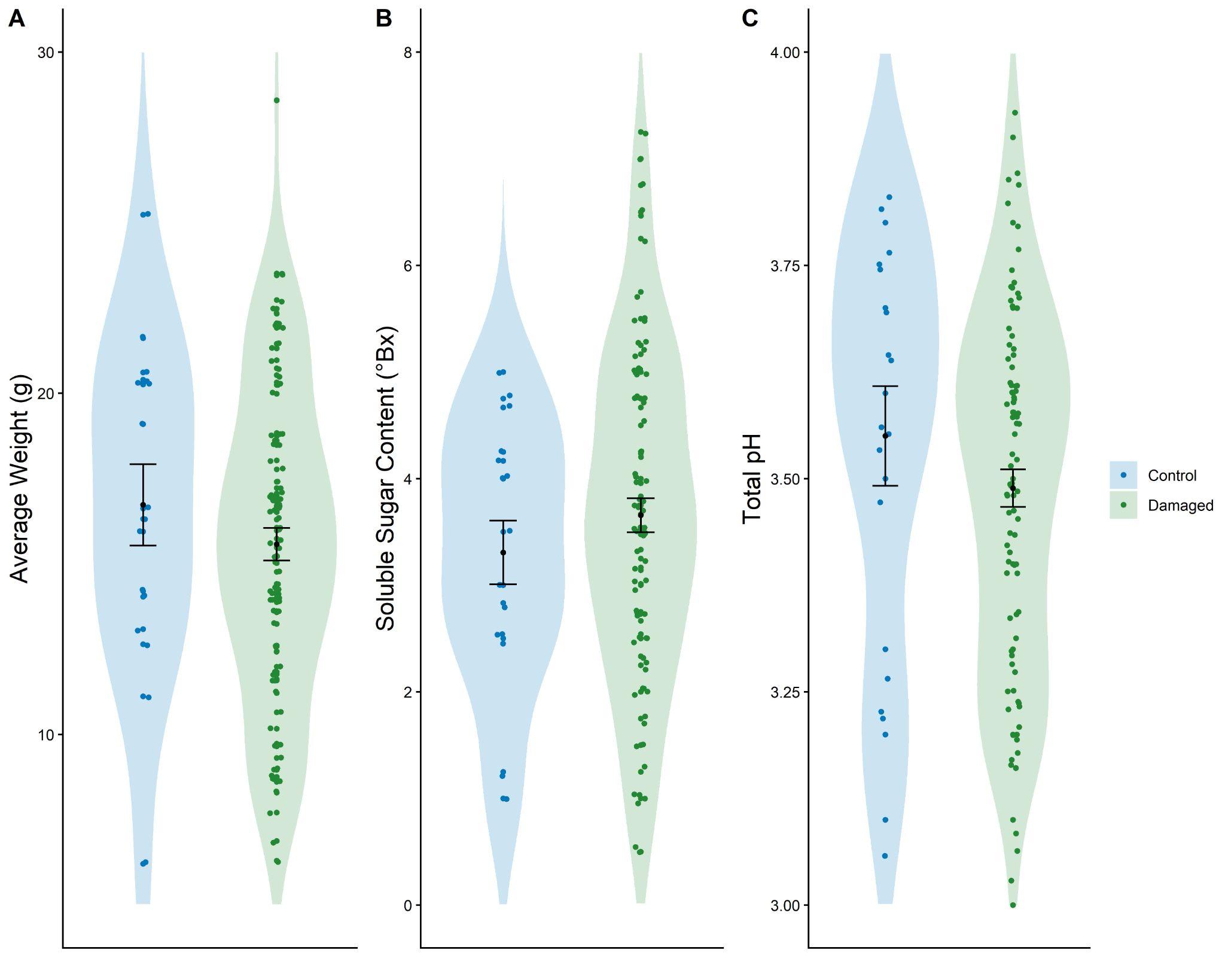


**Figure S2**. Effects of foliar damage on strawberry fruit average weight (A), soluble sugar content (B), and pH (C). Colored points in all figures represent individual strawberry plants (N = 106). Black points and error bars indicate the mean ± standard error.


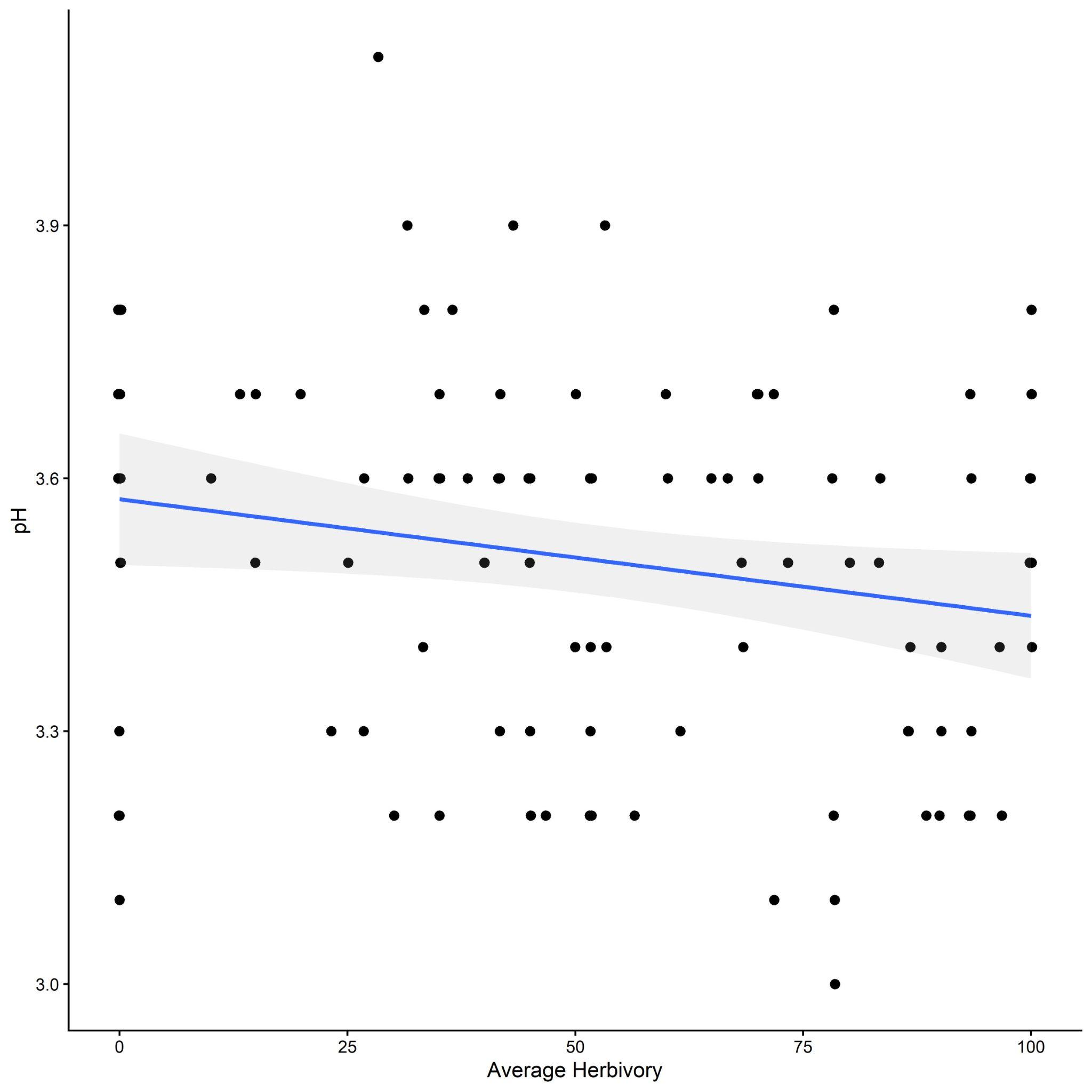


**Figure S3**: Mean fruit pH across average herbivory (% tissue removed). Lines represent the estimated model fits and the grey areas surrounding the lines capture standard deviation around those estimates. Points represent individual plants (N = 106).


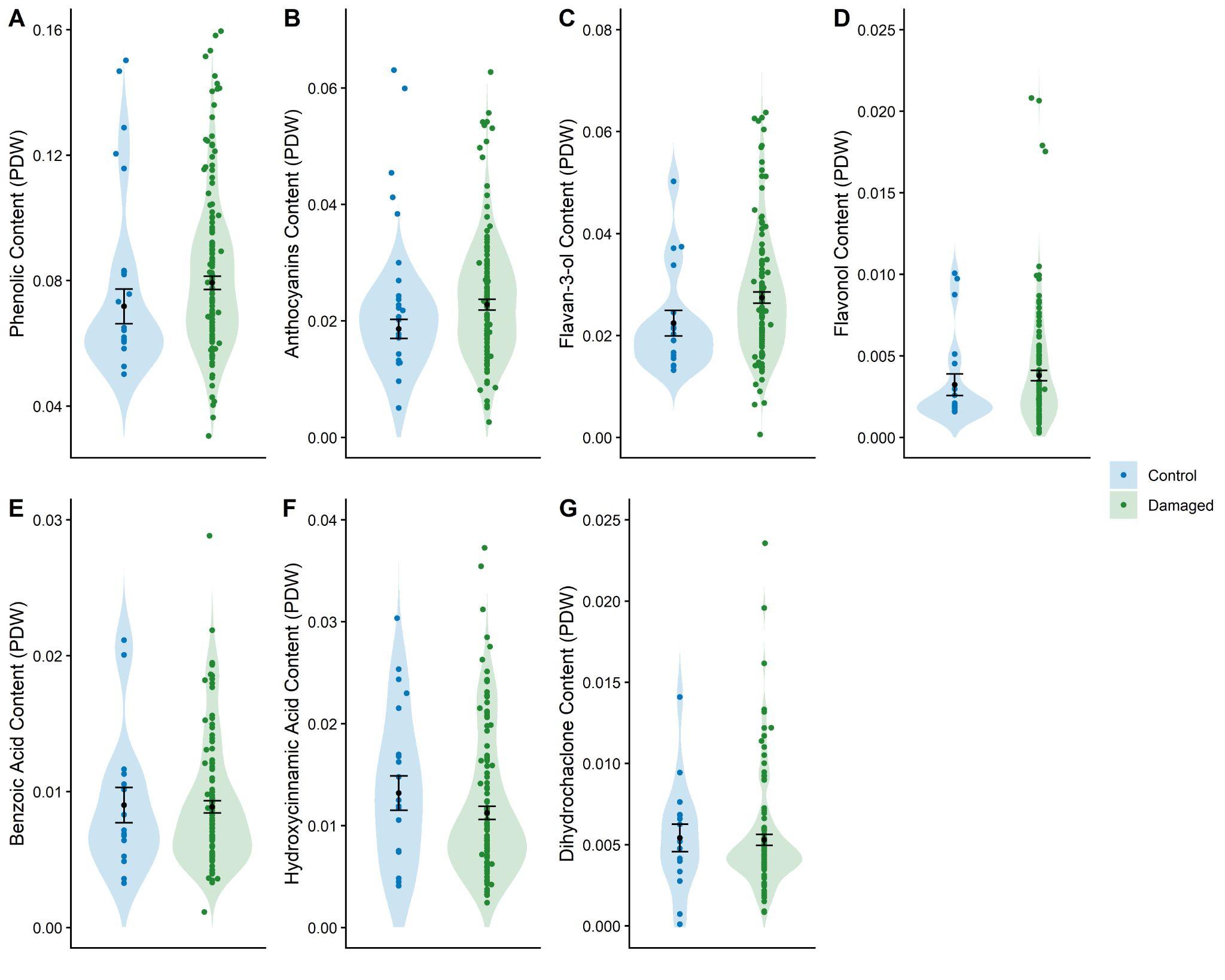


**Figure S4**: Effects of foliar damage on strawberry fruit phenolic content (A), anthocyanin content (B), flavan-3-ol content (C), flavonol content (D), benzoic acid content (E), hydroxycinammic acid content (F), and dihydrochalcone content (G). Colored points in all figures represent individual strawberry plants (N = 106). Black points and error bars indicate the mean ± standard error.


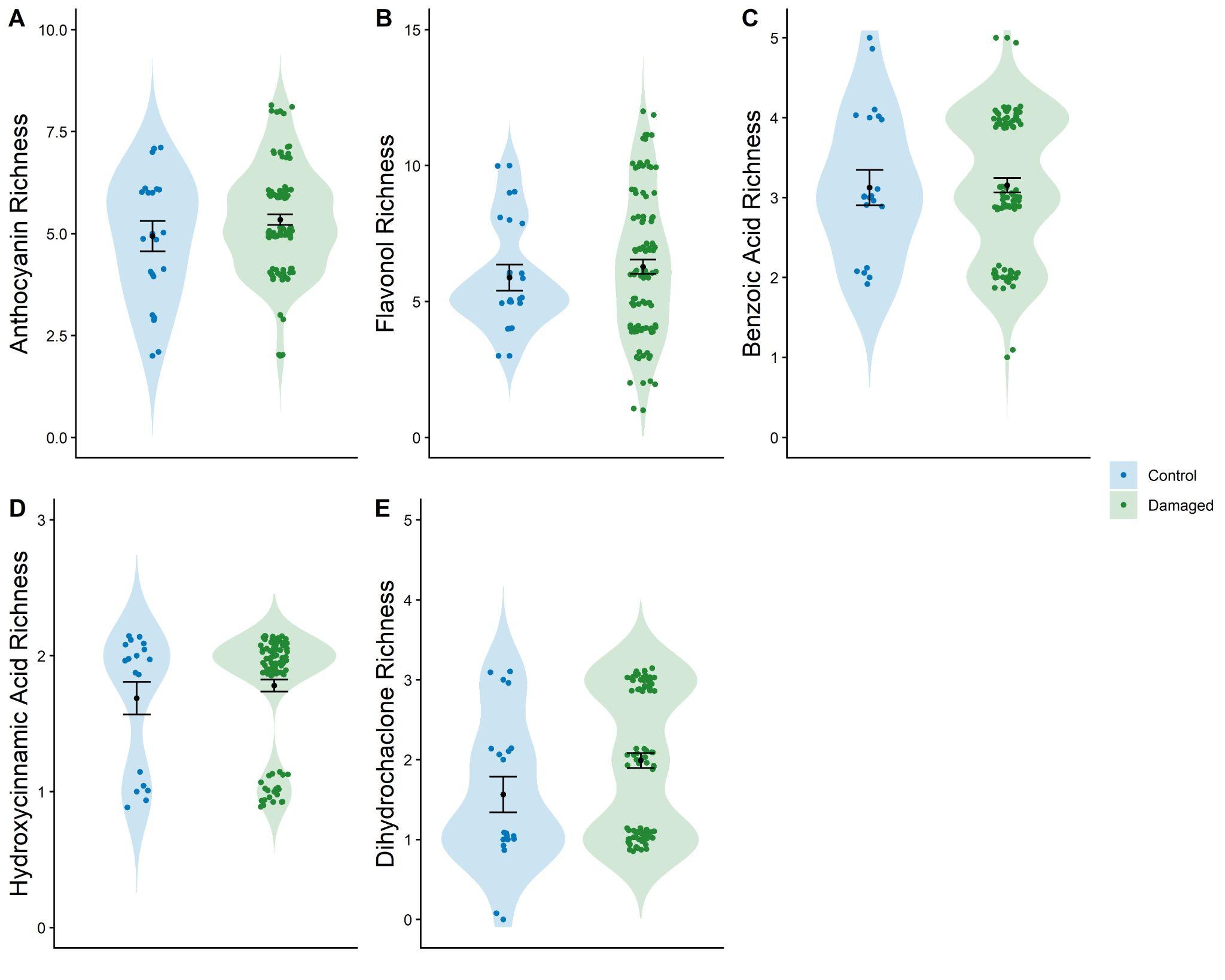


**Figure S5**: Effects of foliar damage on strawberry fruit anthocyanin richness (A), flavonol richness (B), benzoic acid richness (C), hydroxycinammic acid richness (D), and dihydrochalcone richness (E). Colored points in all figures represent individual strawberry plants (N = 106). Black points and error bars indicate the mean ± standard error.


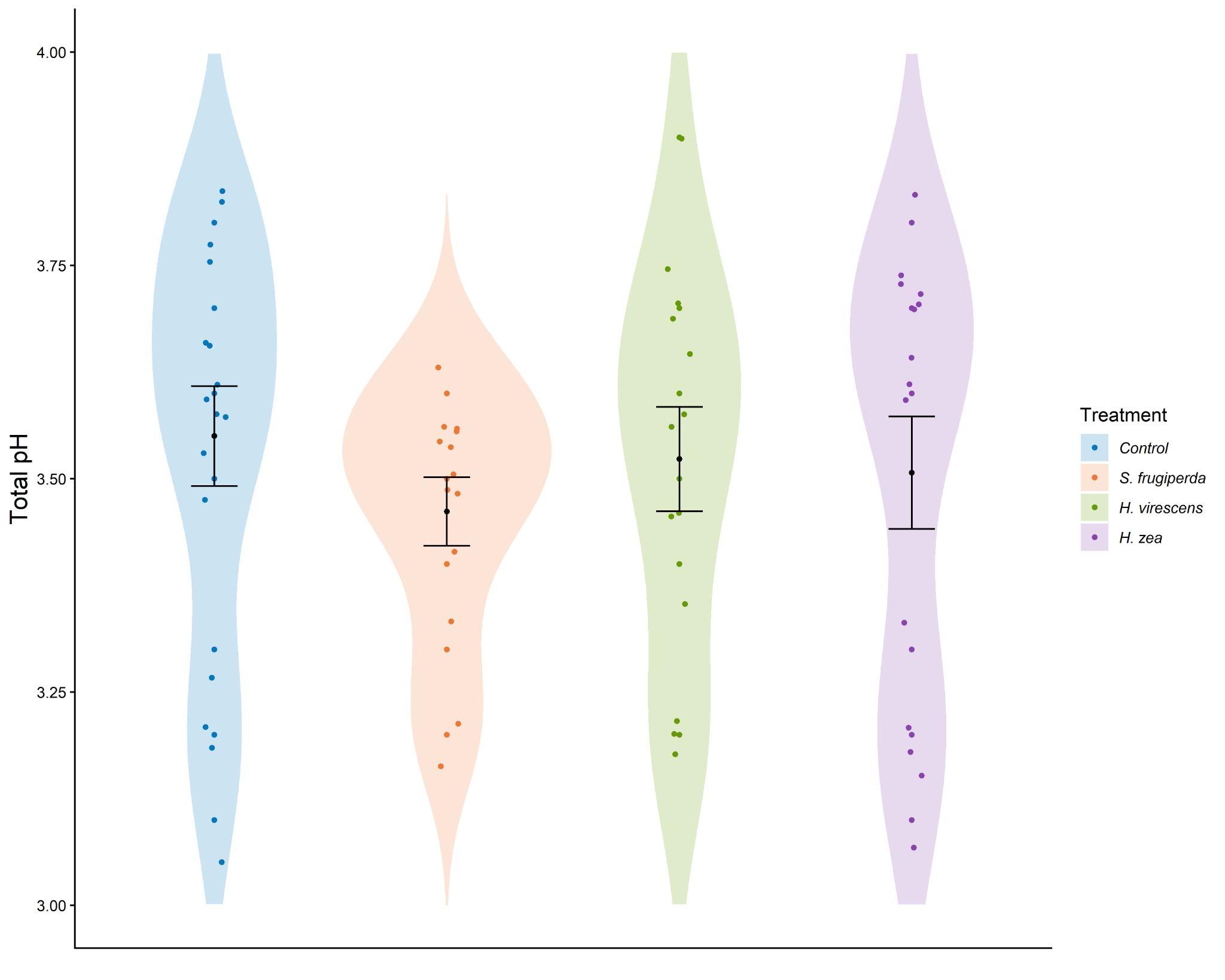


**Figure S6**. Effects of foliar damage from three different herbivores on strawberry fruit pH. Colored points in all figures represent individual strawberry plants (N = 57). Black points and error bars indicate the mean ± standard error.


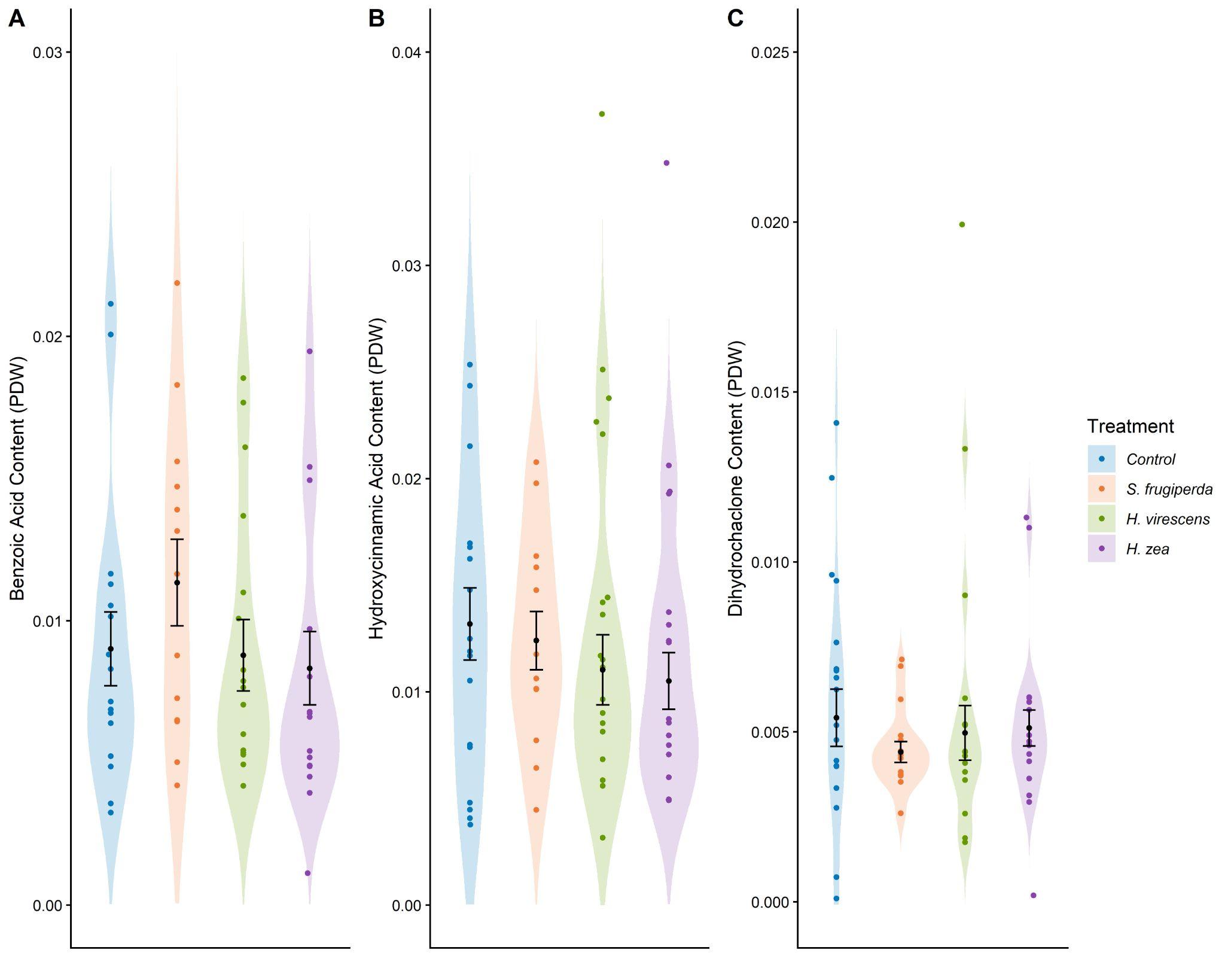


**Figure S7**. Effects of foliar damage from three different herbivores on strawberry fruit benzoic acid content (A), hydroxycinammic acid content (B), and dihydrochalcone content (C). Colored points in all figures represent individual strawberry plants (N = 57). Black points and error bars indicate the mean ± standard error.


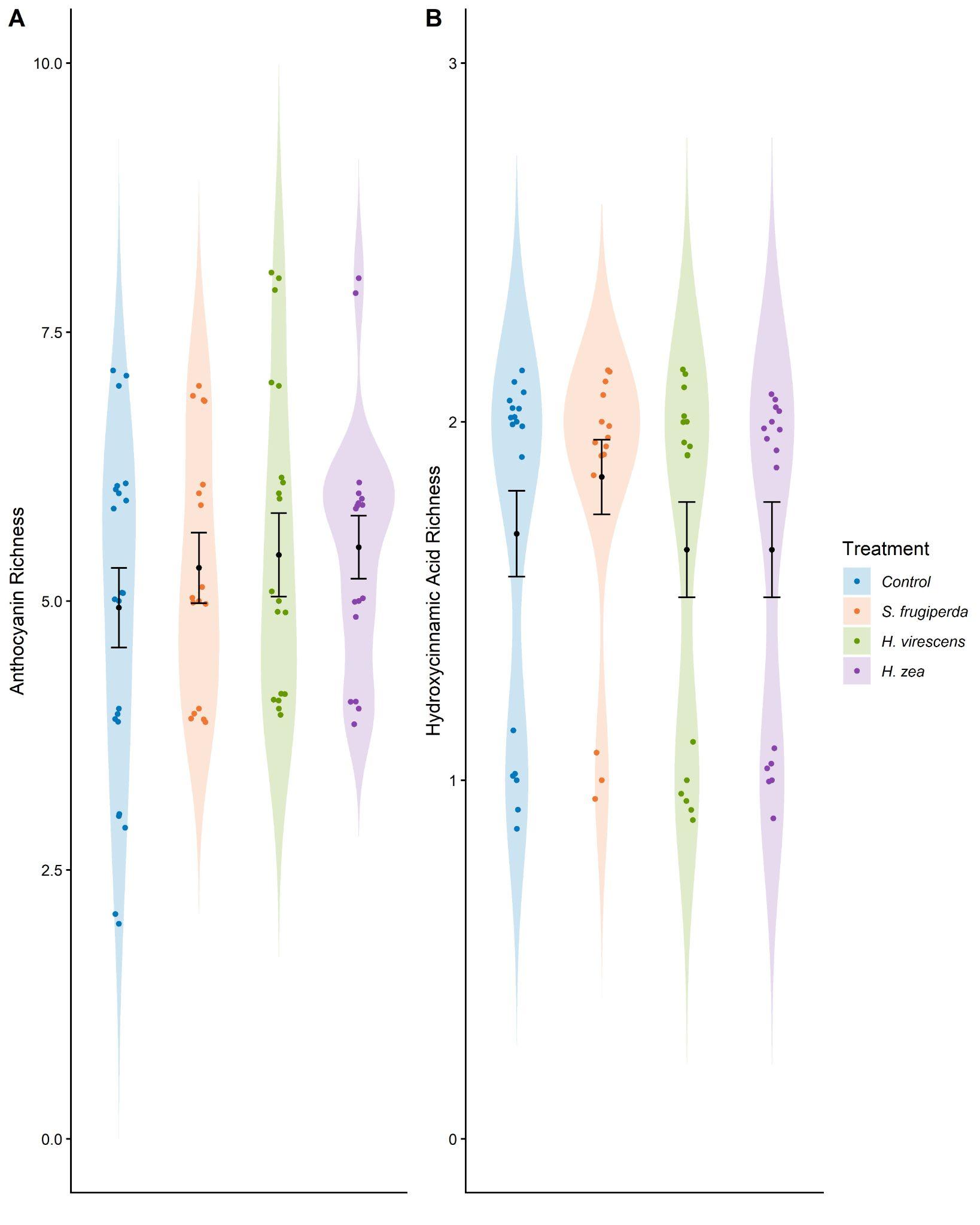


**Figure S8**. Effects of foliar damage from three different herbivores on strawberry fruit anthocyanin richness (A) and hydroxycinammic acid richness (B). Colored points in all figures represent individual strawberry plants (N = 57). Black points and error bars indicate the mean ± standard error.


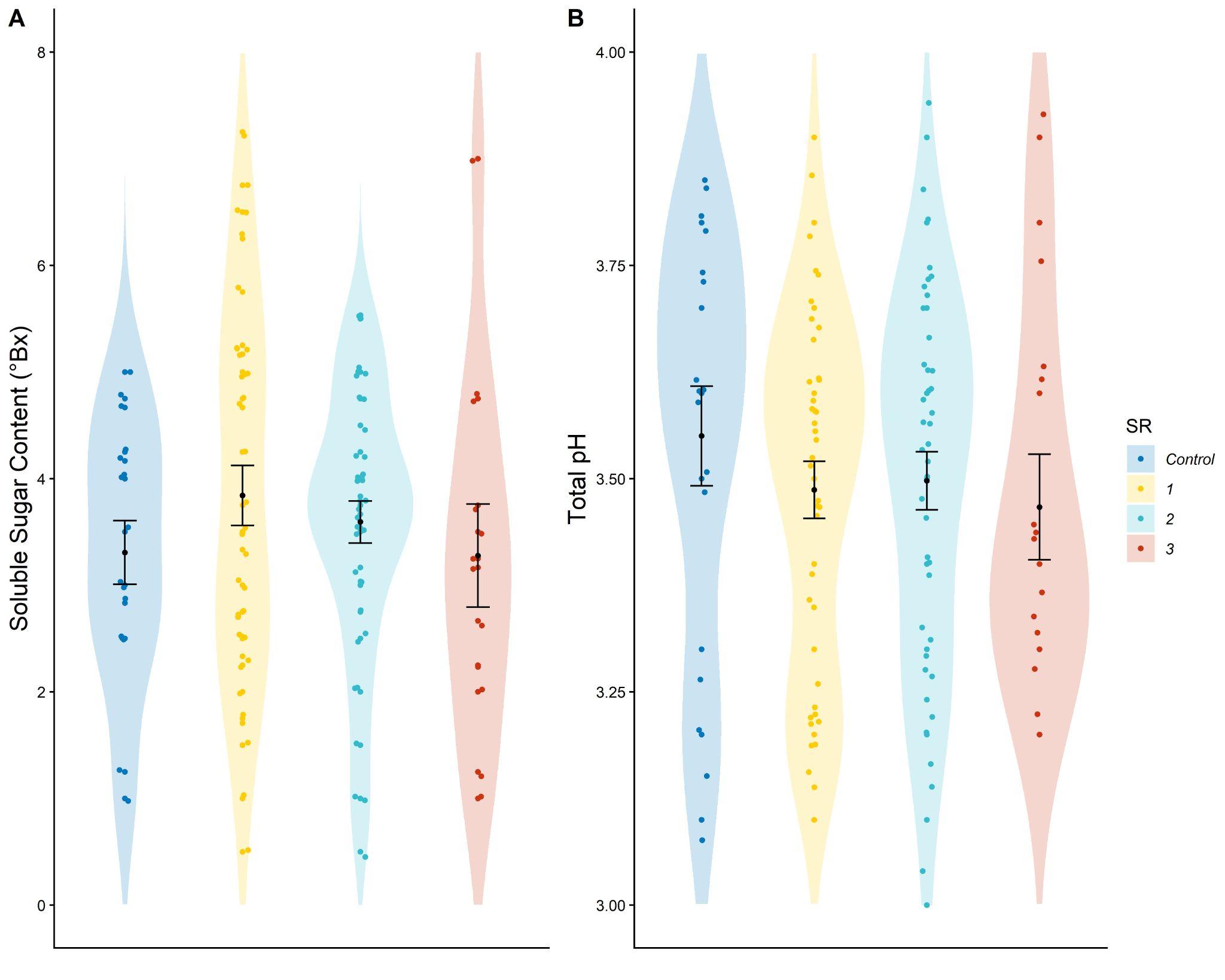


**Figure S9**: Effects of foliar damage from three levels of herbivore species richness on strawberry fruit soluble sugar content (A) and pH (B). Colored points in all figures represent individual strawberry plants (N = 57). Black points and error bars indicate the mean ± standard error.


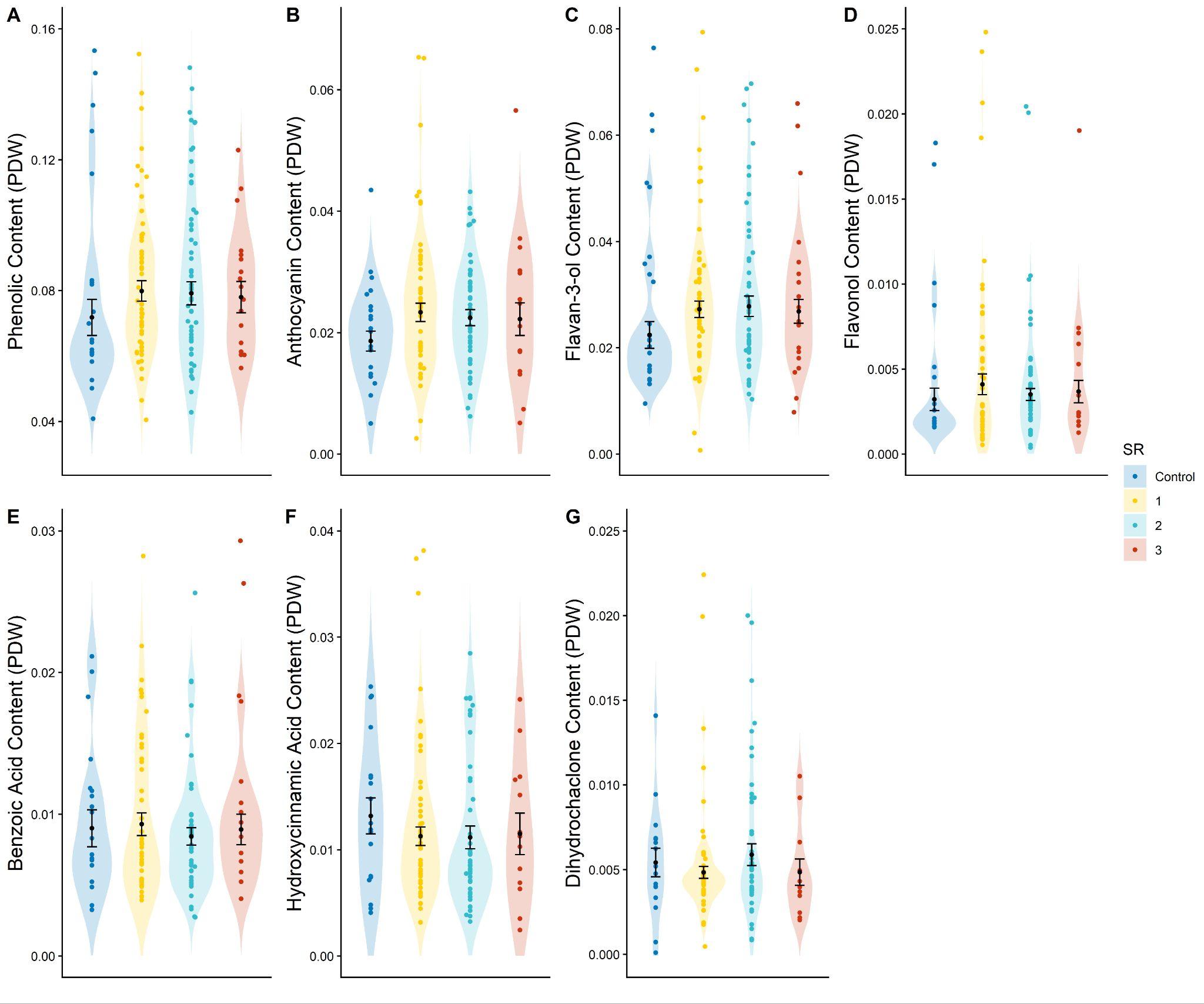


**Figure S10**. Effects of foliar damage from three levels of herbivore species richness on strawberry fruit phenolic content (A), anthocyanin content (B), flavan-3-ol content (C), flavonol content (D), benzoic acid content (E), hydroxycinammic acid content (F), and dihydrochalcone content (G). Colored points in all figures represent individual strawberry plants (N = 107). Black points and error bars indicate the mean ± standard error.


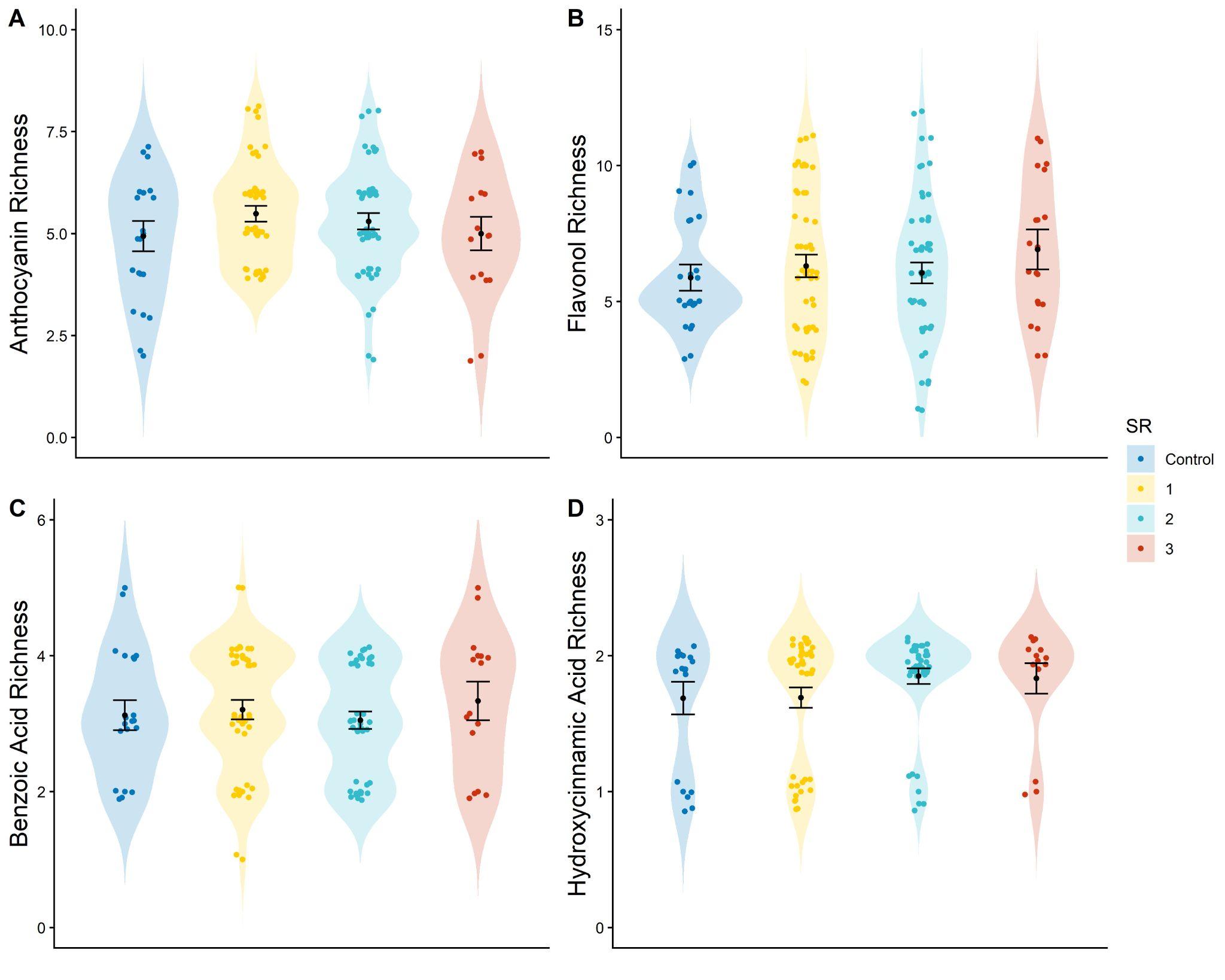


**Figure S11**. Effects of foliar damage from three levels of herbivore species richness on strawberry fruit anthocyanin richness (A), flavonol richness (B), benzoic acid richness (C), and hydroxycinammic acid richness (D), and dihydrochalcone content (G). Colored points in all figures represent individual strawberry plants (N = 107). Black points and error bars indicate the mean ± standard error.

# **Tables**

**Table S1**: Detected compounds, compound classes based on spectra data, and mean (± se) concentration for each treatment (Treatment 1: *Spodoptera frugiperda*; Treatment 2: *Heliothis virescens*; Treatment 3: *Helicoverpa zea*; Treatment 4: *Spodoptera frugiperda* + *Heliothis virescens*; Treatment 5: *Spodoptera frugiperda* + *Helicoverpa zea*; Treatment 6: *Heliothis virescens* + *Helicoverpa zea*; Treatment 7: *Spodoptera frugiperda* + *Heliothis virescens* +

*Helicoverpa zea*)

| **Compound ID** | **Wavelength** | **Compound Class** | **Control** | **Treatment 1** | **Treatment 2** | **Treatment 3** | **Treatment 4** | **Treatment 5** | **Treatment 6** | **Treatment 7** |
| --- | --- | --- | --- | --- | --- | --- | --- | --- | --- | --- |
| B | 280 | Flavanol | 640.40245 | 707.8075 | 770.06326 | 1098.84837 | 661.50185 | 696.07212 | 653.40989 | 639.62145 |
|  |  |  | 77.72079 | 95.34807 | 75.3398 | 265.53548 | 121.90888 | 86.83961 | 88.4331 | 104.99796 |
| C | 280 | Flavanol | 572.1455 | 959.2121 | 666.67029 | 725.49953 | 610.71519 | 556.36499 | 707.90046 | 637.23314 |
|  |  |  | 87.12432 | 326.24215 | 105.88282 | 209.1927 | 94.28613 | 93.53175 | 80.33862 | 121.2323 |
| E | 280 | Benzoic Acid | 264.38703 | 197.29969 | 249.62695 | 324.84465 | 292.35936 | 130.82366 | 356.34903 | 307.49732 |
|  |  |  | 122.35253 | 104.21388 | 109.49958 | 120.94534 | 124.76849 | 89.70823 | 149.62212 | 131.34078 |
| F | 280 | Flavanol | 420.09646 | 728.26359 | 534.03326 | 600.41012 | 357.96702 | 540.79682 | 739.62275 | 501.79247 |
|  |  |  | 108.96671 | 194.81742 | 120.97062 | 133.93067 | 116.03538 | 120.47093 | 85.19739 | 132.88176 |
| H | 320 | Benzoic Acid | 450.7932 | 661.9138 | 462.5371 | 328.9934 | 338.5703 | 462.2365 | 613.7945 | 411.8543 |
|  |  |  | 153.7927 | 181.9153 | 152.2178 | 147.7391 | 146.3137 | 135.4204 | 180.2581 | 147.7107 |
| J | 280 | Flavonol | 191.74322 | 185.62487 | 243.50629 | 105.14649 | 176.25469 | 320.57965 | 229.26941 | 175.5036 |
|  |  |  | 73.29062 | 47.2839 | 116.29407 | 76.91937 | 91.68673 | 119.54774 | 117.12279 | 93.86087 |
| K | 280 | Flavonol | 1256.5918 | 1298.917 | 1127.3238 | 901.9083 | 596.0654 | 1308.7187 | 1246.1011 | 1197.8317 |
|  |  |  | 223.1618 | 175.6556 | 243.6205 | 178.0808 | 149.8661 | 286.5252 | 251.3475 | 271.5473 |
| L | 280 | Flavanol | 481.677 | 385.98646 | 155.7747 | 375.17714 | 304.59893 | 433.25552 | 128.88747 | 548.82868 |
|  |  |  | 203.82054 | 106.1883 | 83.20142 | 170.38391 | 114.11685 | 165.05668 | 86.98587 | 220.07107 |
| M | 280 | Flavonol | 711.43461 | 1127.36854 | 686.24531 | 904.85438 | 879.91629 | 863.68648 | 989.63124 | 816.93626 |
|  |  |  | 194.59929 | 156.00681 | 90.33955 | 209.75897 | 90.71637 | 187.37915 | 169.03714 | 129.2819 |
| N | 280 | Flavonol | 83.80649 | 189.08754 | 55.95519 | 75.45728 | 80.49592 | 220.53556 | 92.6556 | 114.34834 |
|  |  |  | 62.30788 | 82.5384 | 28.2099 | 51.58325 | 37.14742 | 114.41682 | 40.45978 | 42.90491 |
| O | 280 | Flavonol | 117.92832 | 104.64535 | 42.9304 | 123.5529 | 51.29935 | 76.8667 | 120.45374 | 62.88048 |
|  |  |  | 36.72222 | 54.83443 | 21.42433 | 64.24165 | 26.36213 | 29.75729 | 46.23823 | 26.65182 |
| P | 525 | Anthocyanin | 13100.517 | 14137.197 | 16418.598 | 18259.789 | 16261.212 | 15264.944 | 14549.665 | 15559.51 |
|  |  |  | 1196.305 | 2258.626 | 1715.463 | 2017.701 | 1792.428 | 1556.31 | 2152.977 | 2152.626 |
| Q | 525 | Anthocyanin | 800.568 | 814.2837 | 1349.1509 | 1651.7254 | 1424.597 | 1019.9471 | 1407.6387 | 1301.6069 |
|  |  |  | 200.4152 | 295.3444 | 256.8552 | 258.5852 | 250.3366 | 255.8187 | 410.9389 | 350.5429 |
| R | 280 | Flavanol | 722.4918 | 668.1615 | 523.451 | 186.8563 | 326.9007 | 817.5521 | 425.8034 | 620.1998 |
|  |  |  | 203.4198 | 262.0827 | 216.9842 | 130.5316 | 170.9298 | 272.972 | 221.5709 | 202.9495 |
| S | 365 | Anthocyanin | 0 | 38.057429 | 3.329537 | 11.637587 | 4.52803 | 12.911943 | 18.73126 | 38.964555 |
|  |  |  | 0 | 19.957912 | 3.329537 | 8.860407 | 2.57251 | 8.828976 | 12.848707 | 38.295146 |
| U | 365 | Anthocyanin | 38.18008 | 44.08744 | 28.21811 | 50.57799 | 30.33264 | 25.28272 | 22.42822 | 27.59472 |
|  |  |  | 20.25695 | 29.86024 | 13.31754 | 25.81548 | 16.75904 | 18.40453 | 15.05481 | 15.15176 |
| V | 280 | Flavanol | 186.92969 | 245.04581 | 385.95789 | 507.32437 | 430.40166 | 173.17801 | 332.23971 | 458.26406 |
|  |  |  | 128.74922 | 129.41199 | 170.57783 | 257.23034 | 244.95698 | 94.17917 | 228.75456 | 245.05812 |
| W | 280 | Anthocyanin | 194.90418 | 196.29911 | 140.05415 | 158.63567 | 302.22292 | 182.93432 | 131.51513 | 176.97427 |
|  |  |  | 65.36571 | 81.48214 | 53.2413 | 67.47592 | 138.09989 | 59.07882 | 47.39307 | 72.86989 |
| X | 365 | Anthocyanin | 9.809367 | 29.677911 | 9.495569 | 28.723759 | 0 | 50.466853 | 27.711536 | 36.778384 |
|  |  |  | 9.649189 | 29.677911 | 6.677854 | 20.784369 | 0 | 34.801647 | 27.711536 | 36.778384 |
| Y | 280 | Flavanol | 168.97521 | 248.36445 | 141.06909 | 58.42112 | 216.67005 | 136.23396 | 177.5212 | 211.33188 |
|  |  |  | 94.30278 | 133.1306 | 98.29545 | 58.42112 | 216.67005 | 95.58251 | 119.52373 | 110.91182 |
| Z | 280 | Flavanol | 401.6476 | 469.7225 | 254.5734 | 361.0359 | 1204.6699 | 220.309 | 415.3914 | 480.6965 |
|  |  |  | 145.6924 | 213.3955 | 143.0548 | 165.8827 | 902.823 | 118.9299 | 186.3144 | 190.4023 |
| C1 | 525 | Anthocyanin | 840.7331 | 1589.7543 | 1333.3604 | 1357.856 | 1643.4147 | 1973.0606 | 1335.8205 | 1081.9255 |
|  |  |  | 325.9687 | 424.7929 | 250.0143 | 343.0009 | 578.7924 | 427.0885 | 521.9599 | 325.3188 |
| C3 | 525 | Anthocyanin | 869.4815 | 708.0644 | 1478.6069 | 740.6476 | 485.0857 | 609.8505 | 835.0481 | 825.882 |
|  |  |  | 250.3031 | 497.9437 | 501.7864 | 299.3478 | 279.4603 | 281.9528 | 385.11 | 349.3765 |
| C4 | 525 | Anthocyanin | 1397.0133 | 1686.8908 | 851.3385 | 662.7299 | 815.3334 | 1067.085 | 859.7459 | 1175.0624 |
|  |  |  | 520.3271 | 355.5086 | 351.0762 | 268.2291 | 248.1104 | 416.1882 | 341.497 | 394.5544 |
| C6 | 280 | Flavanol | 415.7716 | 441.7689 | 107.7855 | 469.2774 | 178.6608 | 189.3958 | 230.7231 | 645.004 |
|  |  |  | 314.0022 | 207.0106 | 107.7855 | 213.8851 | 120.6544 | 137.4905 | 230.7231 | 291.8574 |
| C7 | 280 | Flavanol | 917.9007 | 1434.4185 | 796.349 | 988.6306 | 1300.0957 | 1229.8282 | 977.3813 | 914.1185 |
|  |  |  | 192.213 | 409.9272 | 213.3697 | 241.7742 | 361.5305 | 272.5529 | 341.6268 | 262.1494 |
| C8 | 280 | Flavanol | 392.8853 | 1268.2643 | 543.0052 | 466.5624 | 1379.6054 | 1065.4567 | 1165.8387 | 1325.767 |
|  |  |  | 225.5314 | 359.0337 | 366.3046 | 217.4812 | 693.4108 | 439.3882 | 571.2376 | 432.9131 |
| C9 | 280 | Flavanol | 830.9623 | 1899.5339 | 1127.2144 | 1023.964 | 581.6904 | 598.0974 | 803.3537 | 1017.9087 |
|  |  |  | 397.9965 | 598.8984 | 489.6534 | 535.8125 | 276.7024 | 341.6503 | 467.5601 | 456.2022 |
| C10 | 320 | Flavanol | 1562.2794 | 2031.1529 | 1394.1759 | 1310.123 | 1172.5969 | 1357.5032 | 1511.6358 | 1174.817 |
|  |  |  | 453.503 | 607.0491 | 277.8014 | 207.0239 | 340.8146 | 352.4906 | 405.8743 | 355.5441 |
| C11 | 280 | Flavonol | 153.08326 | 256.72784 | 88.66077 | 92.96834 | 143.89758 | 206.12982 | 122.35715 | 132.19471 |
|  |  |  | 77.5136 | 90.81956 | 55.1969 | 53.52648 | 56.47003 | 80.75309 | 55.85455 | 46.78279 |
| C12 | 280 | Flavonol | 63.120681 | 119.0471 | 200.339291 | 31.712779 | 3.219262 | 63.628288 | 51.990497 | 201.554581 |
|  |  |  | 36.276845 | 87.967794 | 88.252768 | 17.778803 | 3.219262 | 53.913457 | 28.833597 | 105.772308 |
| C13 | 280 | Flavonol | 241.88986 | 640.26923 | 189.10419 | 386.67455 | 411.08778 | 371.28418 | 404.06168 | 320.90512 |
|  |  |  | 84.11809 | 130.15297 | 59.52019 | 116.82285 | 109.25012 | 94.61828 | 122.34675 | 125.51187 |
| C14 | 280 | Flavanol | 1028.2288 | 791.6425 | 926.9645 | 1127.377 | 958.7874 | 1298.4325 | 692.5987 | 962.0742 |
|  |  |  | 202.8458 | 192.2706 | 271.4707 | 297.5351 | 249.9215 | 338.183 | 272.6091 | 326.4224 |
| C15 | 280 | Flavonol | 57.64726 | 320.79916 | 33.69628 | 157.75113 | 58.99489 | 61.95924 | 65.83141 | 107.57256 |
|  |  |  | 27.90103 | 87.29577 | 23.41112 | 78.27562 | 28.19103 | 36.27823 | 52.22807 | 45.63248 |
| C16 | 320 | Flavonol | 223.03455 | 1562.48005 | 174.13856 | 218.38816 | 216.69142 | 252.83001 | 227.98663 | 353.44669 |
|  |  |  | 76.8502 | 1148.31534 | 80.98737 | 100.53872 | 95.72409 | 64.29758 | 102.4628 | 121.23644 |
| C17 | 280 | Benzoic Acid | 6235.6289 | 8007.2701 | 6001.1757 | 5851.5809 | 5339.8174 | 5430.5113 | 6068.2913 | 5903.0435 |
|  |  |  | 1045.3335 | 1379.235 | 1044.6652 | 1041.633 | 712.1883 | 840.0546 | 1135.4004 | 797.3917 |
| C18 | 280 | Dihydrochalcone | 4778.5422 | 3752.8339 | 3935.8273 | 3681.6619 | 4162.0041 | 5597.2983 | 5458.2757 | 4017.9933 |
|  |  |  | 898.7391 | 438.3742 | 899.15 | 497.6418 | 922.8356 | 1185.2059 | 1767.8032 | 808.3755 |
| C19 | 280 | Flavanol | 1363.3198 | 1137.5057 | 699.8659 | 714.0917 | 803.4992 | 1290.2249 | 1627.2204 | 765.9509 |
|  |  |  | 991.2131 | 471.8399 | 403.4172 | 342.7068 | 296.5325 | 590.0905 | 1049.1458 | 306.7645 |
| C20 | 280 | Flavanol | 1389.7549 | 2308.1473 | 1517.6406 | 1220.8201 | 1337.6243 | 1451.3703 | 1863.1119 | 1681.6286 |
|  |  |  | 381.1021 | 533.7942 | 365.1837 | 341.4249 | 322.0029 | 425.3025 | 415.4593 | 234.4018 |
| C21 | 280 | Flavanol | 1299.5293 | 1892.928 | 994.9576 | 1601.4355 | 929.2115 | 1286.941 | 1566.0061 | 737.1862 |
|  |  |  | 277.0498 | 475.1865 | 258.2856 | 267.2731 | 210.4634 | 300.255 | 502.3687 | 197.6009 |
| C22 | 280 | Flavanol | 290.71322 | 193.56357 | 242.04233 | 452.17805 | 418.5233 | 159.85114 | 0 | 266.99415 |
|  |  |  | 143.487 | 108.04511 | 108.61366 | 162.77546 | 219.44313 | 86.91961 | 0 | 185.08821 |
| C23 | 280 | Flavanol | 155.38257 | 254.7187 | 374.44945 | 1129.63433 | 315.41909 | 311.68261 | 441.9324 | 434.65703 |
|  |  |  | 69.64611 | 115.39718 | 161.77931 | 436.63135 | 96.32263 | 112.31357 | 144.91286 | 251.35316 |
| C24 | 280 | Flavanol | 1543.8547 | 2186.3538 | 2256.6267 | 2761.8799 | 2165.8235 | 1911.8519 | 2168.543 | 1932.6272 |
|  |  |  | 314.1222 | 476.4332 | 233.7927 | 471.7988 | 239.0817 | 271.6564 | 455.572 | 403.1512 |
| C25 | 280 | Flavanol | 288.5833 | 492.8961 | 474.4725 | 821.0035 | 515.3615 | 591.7212 | 374.4459 | 478.8779 |
|  |  |  | 138.297 | 164.6482 | 132.5516 | 289.2308 | 209.2067 | 184.1914 | 193.5065 | 181.5 |
| C26 | 280 | Flavanol | 168.1209 | 491.1068 | 681.3511 | 728.9054 | 705.9721 | 298.8742 | 578.2981 | 852.1223 |
|  |  |  | 115.1952 | 207.4968 | 172.8828 | 310.0367 | 274.1872 | 161.0863 | 177.0258 | 238.7261 |
| C27 | 365 | Flavonol | 0 | 36.14935 | 0 | 53.87424 | 0 | 16.40511 | 13.38006 | 40.81706 |
|  |  |  | 0 | 21.40223 | 0 | 38.61849 | 0 | 14.56201 | 13.38006 | 27.16387 |
| C28 | 280 | Flavanol | 47.50019 | 208.42255 | 164.11056 | 111.75132 | 202.41769 | 255.79094 | 303.2338 | 279.99395 |
|  |  |  | 47.50019 | 91.09828 | 90.59359 | 75.98211 | 108.15609 | 98.97182 | 129.78763 | 124.63378 |
| C30 | 280 | Flavanol | 167.1776 | 348.27499 | 372.04508 | 583.67843 | 733.76097 | 437.66121 | 551.50652 | 584.9539 |
|  |  |  | 93.48277 | 170.11897 | 149.02237 | 175.95717 | 219.15702 | 135.59518 | 209.24261 | 157.91216 |
| C31 | 280 | Flavanol | 338.4635 | 507.1421 | 188.7404 | 270.196 | 595.8684 | 565.4519 | 363.2231 | 650.0346 |
|  |  |  | 105.6154 | 120.8926 | 102.698 | 103.5631 | 285.4118 | 140.5214 | 158.5805 | 235.9246 |
| C32 | 280 | Flavonol | 0.9347209 | 10.5585744 | 0 | 1.4562065 | 0 | 0.49485 | 6.1951808 | 0 |
|  |  |  | 0.9347209 | 10.3984105 | 0 | 1.4562065 | 0 | 0.49485 | 6.1951808 | 0 |
| C33 | 280 | Flavanol | 1115.45986 | 768.29588 | 1260.39379 | 1182.10472 | 866.51294 | 1158.33643 | 1269.40464 | 1178.354 |
|  |  |  | 167.31018 | 184.20806 | 202.91383 | 67.04304 | 159.88189 | 189.14642 | 255.45461 | 204.25303 |
| C34 | 280 | Flavanol | 318.7136 | 708.876 | 613.255 | 571.1926 | 666.7646 | 483.3685 | 654.3406 | 0 |
|  |  |  | 149.2647 | 169.2265 | 255.6846 | 209.9858 | 208.2135 | 197.5711 | 289.6144 | 0 |
| C35 | 280 | Flavanol | 1171.9913 | 1730.9362 | 1028.2524 | 899.0322 | 1053.3826 | 1258.0587 | 1390.0859 | 1629.3751 |
|  |  |  | 250.7731 | 330.5209 | 209.8154 | 234.412 | 254.1591 | 307.9784 | 457.9686 | 431.8568 |
| C36 | 280 | Flavanol | 578.3296 | 765.69642 | 543.09349 | 449.01458 | 647.13437 | 526.42075 | 472.40754 | 724.90152 |
|  |  |  | 111.29535 | 140.29947 | 141.06063 | 98.89312 | 112.20407 | 107.42781 | 114.73194 | 169.11983 |
| C37 | 280 | Dihydrochalcone | 420.7042 | 298.5475 | 503.3185 | 868.2083 | 527.793 | 363.5482 | 498.8377 | 385.7386 |
|  |  |  | 129.1108 | 130.6319 | 142.1962 | 103.4673 | 135.9256 | 121.7536 | 147.642 | 148.0187 |
| Caffeic Acid | 280 | Benzoic Acid | 668.3571 | 996.4038 | 588.8791 | 417.4964 | 468.2508 | 386.8221 | 552.967 | 732.3546 |
|  |  |  | 172.1923 | 176.3643 | 181.807 | 178.1683 | 243.3985 | 173.0495 | 235.5001 | 234.8576 |
| Cyanidin Glucoside | 525 | Anthocyanin | 1258.3196 | 1732.634 | 1873.2262 | 2454.1748 | 2385.0243 | 1625.6702 | 2330.0448 | 1895.2692 |
|  |  |  | 241.5787 | 348.1311 | 314.895 | 300.9886 | 286.6165 | 300.3818 | 555.745 | 337.4186 |
| Catechin | 280 | Flavanol | 3061.1455 | 4633.9025 | 3756.2448 | 4332.3049 | 4263.7503 | 6894.4647 | 4745.9747 | 4004.0964 |
|  |  |  | 572.5173 | 390.9935 | 227.0923 | 813.5908 | 249.3517 | 2811.1268 | 485.0397 | 287.8675 |
| Chlorogenic Acid | 320 | Hydroxycinaminic Acid | 12423.815 | 11451.543 | 10437.207 | 9876.094 | 8941.758 | 11042.002 | 10898.945 | 10748.661 |
|  |  |  | 1607.175 | 1263.444 | 1565.665 | 1241.419 | 1782.622 | 1896.278 | 2074.836 | 1886.005 |
| Epicatechin | 280 | Flavanol | 275.16934 | 346.55819 | 414.47694 | 264.1068 | 83.56808 | 400.89694 | 83.90739 | 410.62916 |
|  |  |  | 152.81259 | 138.98999 | 189.28434 | 151.99599 | 83.56808 | 172.39168 | 83.90739 | 175.69278 |
| Ferulic Acid | 320 | Hydroxycinaminic Acid | 657.8218 | 851.3807 | 496.8138 | 537.5037 | 540.9466 | 763.3507 | 751.4595 | 641.1824 |
|  |  |  | 150.5147 | 154.0495 | 114.8425 | 144.2058 | 110.225 | 124.2686 | 145.5793 | 105.0196 |
| Avicularin | 365 | Flavonol | 22.5241 | 118.557 | 18.04144 | 158.25064 | 36.85064 | 57.44109 | 117.17741 | 52.73691 |
|  |  |  | 11.12809 | 53.93946 | 12.60048 | 92.67017 | 16.91618 | 35.24928 | 73.22 | 23.04332 |
| Phloridzin | 280 | Dihydrochalcone | 117.24698 | 257.28942 | 431.11561 | 462.59277 | 402.02689 | 151.5551 | 281.409 | 350.57654 |
|  |  |  | 63.04666 | 94.68731 | 89.51979 | 97.34737 | 103.19172 | 81.63236 | 118.98348 | 106.04637 |

**Table S2**: Generalized linear mixed model outputs showing the effects of treatments on fruit quality (soluble sugar content, average weight, pH), phenolic content and content by subclass (anthocyanins, flavonols, flavan-3-ols, benzoic acids, dihydrochalcones, and hydroxycinnamic acids), and phenolic richness and richness by subclass (anthocyanins, flavonols, flavan-3-ols, benzoic acids, dihydrochalcones, and hydroxycinnamic acids).

| **Response Variable** | **Model Distribution** | **Fixed Effects** | **χ2** | **df** | **p** |
| --- | --- | --- | --- | --- | --- |
| Average Herbivory | Beta |  |  |  |  |
|  |  | Treatment | 13.711 | 6 | 0.03304 |
| Soluble Sugar Content | Gaussian |  |  |  |  |
|  |  | Treatment | 16.5043 | 7 | 0.02089 |
|  |  | Average Herbivory | 0.0133 | 1 | 0.90814 |
| Average Weight | Gaussian |  |  |  |  |
|  |  | Treatment | 14.6689 | 7 | 0.04049 |
|  |  | Average Herbivory | 1.5804 | 1 | 0.2087 |
| pH | Gaussian |  |  |  |  |
|  |  | Treatment | 4.2188 | 7 | 0.75426 |
|  |  | Average Herbivory | 3.6456 | 1 | 0.05622 |
| Phenolic Content | Beta |  |  |  |  |
|  |  | Treatment | 5.4143 | 7 | 0.6095 |
|  |  | Average Herbivory | 0.2751 | 1 | 0.5999 |
| Anthocyanin Content | Beta |  |  |  |  |
|  |  | Treatment | 5.5047 | 7 | 0.5986 |
|  |  | Average Herbivory | 0.0096 | 1 | 0.9221 |
| Flavan-3-ol Content | Beta |  |  |  |  |
|  |  | Treatment | 7.431 | 7 | 0.3854 |
|  |  | Average Herbivory | 0.0041 | 1 | 0.949 |
| Flavonol Content | Beta |  |  |  |  |
|  |  | Treatment | 10.4788 | 7 | 0.163 |
|  |  | Average Herbivory | 0.5286 | 1 | 0.4672 |
| Benzoic Acid Content | Beta |  |  |  |  |
|  |  | Treatment | 6.1816 | 7 | 0.5187 |
|  |  | Average Herbivory | 0.9531 | 1 | 0.3289 |
| Dihydrochaclone Content | Beta |  |  |  |  |
|  |  | Treatment | 1.477 | 7 | 0.9831 |
|  |  | Average Herbivory | 0.0469 | 1 | 0.8285 |
| Hydroxycinnamic Acid Content | Beta |  |  |  |  |
|  |  | Treatment | 2.7375 | 7 | 0.9082 |
|  |  | Average Herbivory | 0.0015 | 1 | 0.9694 |
| Phenolic Richness | Gaussian |  |  |  |  |
|  |  | Treatment | 10.1166 | 7 | 0.1821 |
|  |  | Average Herbivory | 1.1262 | 1 | 0.2886 |
| Anthocyanin Richness | Gaussian |  |  |  |  |
|  |  | Treatment | 2.634 | 7 | 0.9167 |
|  |  | Average Herbivory | 0.1058 | 1 | 0.745 |
| Flavan-3-ol Richness | Gaussian |  |  |  |  |
|  |  | Treatment | 7.8062 | 7 | 0.35 |
|  |  | Average Herbivory | 0.3157 | 1 | 0.5742 |
| Flavonol Richness | Gaussian |  |  |  |  |
|  |  | Treatment | 8.3816 | 7 | 0.3001 |
|  |  | Average Herbivory | 1.0669 | 1 | 0.3016 |
| Benzoic Acid Richness | Gaussian |  |  |  |  |
|  |  | Treatment | 9.1889 | 7 | 0.23937 |
|  |  | Average Herbivory | 2.726 | 1 | 0.09873 |
| Dihydrochaclone Richness | Gaussian |  |  |  |  |
|  |  | Treatment | 14.4102 | 7 | 0.04435 |
|  |  | Average Herbivory | 0.3948 | 1 | 0.52977 |
| Hydroxycinnamic Acid Richness | Gaussian |  |  |  |  |
|  |  | Treatment | 6.2562 | 7 | 0.5102 |
|  |  | Average Herbivory | 0.1531 | 1 | 0.6956 |

**Table S3**: Planned contrasts evaluating the effect of species identity (H2) and species richness (H3) on average herbivory. Sf: *Spodoptera frugiperda*; Hv: *Heliothis virescens*; Hz: *Helicoverpa zea*; SR1: Treatments 1, 2, and 3; SR2: Treatments 4, 5, and 6; SR3: Treatment 7.

|  | **Planned Contrasts** | **contrast** | **estimate** | **df** | **t.ratio** | **p.value** |
| --- | --- | --- | --- | --- | --- | --- |
| **Response variable: Average Herbivory** | | |  |  |  |  |
|  | ***Effects of species identity (H2)*** | |  |  |  |  |
|  | Sf v Hv | 0.69 | 0.301 | 1 | -0.849 | 0.3956 |
|  | Sf v Hz | 2.68 | 1.18 | 1 | 2.238 | 0.0252 |
|  | Hv v Hz | 3.88 | 1.68 | 1 | 3.14 | 0.0017 |
|  | ***Effects of herbivore species richness (H3)*** | | |  |  |  |
|  | SR1 v SR2 | 1.352 | 0.341 | 1 | 1.195 | 0.2319 |
|  | SR1 v SR3 | 0.74 | 0.274 | 1 | -0.814 | 0.4157 |
|  | SR2 v SR3 | 0.548 | 0.204 | 1 | -1.615 | 0.1063 |

**Table** S4: Planned contrasts evaluating the effect of herbivore damage (H1), species identity (H2), and species richness (H3) on fruit quality (soluble sugar content, average weight, pH). Sf: *Spodoptera frugiperda*; Hv: *Heliothis virescens*; Hz: *Helicoverpa zea*; SR1: Treatments 1, 2, and 3; SR2: Treatments 4, 5, and 6; SR3: Treatment 7.

| **Response Variable** | **Planned contrasts** | **estimate** | **SE** | **df** | ***t*** | ***p*** |
| --- | --- | --- | --- | --- | --- | --- |
| **Soluble Sugar Content** | |  |  |  |  |  |
|  | ***Overall effects of damage (H1)*** | |  |  |  |  |
|  | C v D | -0.292 | 0.514 | 95 | -0.567 | 0.572 |
|  | ***Effects of species identity (H2)*** | |  |  |  |  |
|  | Sf v Hv | -1.447 | 0.536 | 95 | -2.7 | 0.0082 |
|  | Sf v Hz | -1.884 | 0.536 | 95 | -3.513 | 0.0007 |
|  | Hv v Hz | -0.437 | 0.539 | 95 | -0.81 | 0.4201 |
|  | Sf v C | 0.641 | 0.641 | 95 | 0.999 | 0.3202 |
|  | Hv v C | -0.806 | 0.65 | 95 | -1.24 | 0.218 |
|  | Hz v C | -1.243 | 0.576 | 95 | -2.16 | 0.0333 |
|  | ***Effects of herbivore species richness (H3)*** | | |  |  |  |
|  | SR1 v SR2 | 0.2344 | 0.311 | 95 | 0.753 | 0.4533 |
|  | SR1 v SR3 | 0.5438 | 0.451 | 95 | 1.207 | 0.2304 |
|  | SR2 v SR3 | 0.3094 | 0.455 | 95 | 0.679 | 0.4985 |
|  | SR1 v C | -0.4697 | 0.541 | 95 | -0.869 | 0.3871 |
|  | SR2 v C | -0.2353 | 0.533 | 95 | -0.441 | 0.66 |
|  | SR3 v C | 0.0741 | 0.649 | 95 | 0.114 | 0.9094 |
| **Average Weight** | |  |  |  |  |  |
|  | ***Overall effects of damage (H1)*** | |  |  |  |  |
|  | C v D |  |  |  |  |  |
|  | ***Effects of species identity (H2)*** | |  |  |  |  |
|  | Sf v Hv | 2.56 | 1.61 | 96 | 1.593 | 0.1145 |
|  | Sf v Hz | 3.2 | 1.65 | 96 | 1.936 | 0.0558 |
|  | Hv v Hz | 3.371 | 1.68 | 96 | 2.004 | 0.0479 |
|  | Sf v C | 0.17 | 1.67 | 96 | 0.102 | 0.919 |
|  | Hv v C | -0.875 | 2.01 | 96 | -0.436 | 0.6637 |
|  | Hz v C | 2.326 | 2.03 | 96 | 1.143 | 0.2558 |
|  | ***Effects of herbivore species richness (H3)*** | | |  |  |  |
|  | SR1 v SR2 | 1.96 | 0.972 | 96 | 2.015 | 0.0467 |
|  | SR1 v SR3 | 2.87 | 1.41 | 96 | 2.036 | 0.0445 |
|  | SR2 v SR3 | 0.91 | 1.43 | 96 | 0.637 | 0.5255 |
|  | SR1 v C | 1.32 | 1.7 | 96 | 0.776 | 0.4396 |
|  | SR2 v C | 3.27 | 1.67 | 96 | 1.964 | 0.0524 |
|  | SR3 v C | 4.18 | 2.03 | 96 | 2.06 | 0.0421 |
| **pH** | |  |  |  |  |  |
|  | ***Overall effects of damage (H1)*** | |  |  |  |  |
|  | C v D | -0.0491 | 0.0787 | 95 | -0.624 | 0.5341 |
|  | ***Effects of species identity (H2)*** | |  |  |  |  |
|  | Sf v Hv | -0.0719 | 0.0819 | 95 | -0.878 | 0.3824 |
|  | Sf v Hz | -0.0153 | 0.0818 | 95 | -0.187 | 0.8522 |
|  | Hv v Hz | 0.0566 | 0.083 | 95 | 0.682 | 0.4968 |
|  | Sf v C | -0.0249 | 0.0978 | 95 | -0.254 | 0.7997 |
|  | Hv v C | -0.0968 | 0.101 | 95 | -0.957 | 0.3412 |
|  | Hz v C | -0.0402 | 0.0877 | 95 | -0.458 | 0.6481 |
|  | ***Effects of herbivore species richness (H3)*** | | |  |  |  |
|  | SR1 v SR2 | 0.00295 | 0.0475 | 95 | 0.062 | 0.9506 |
|  | SR1 v SR3 | 0.025 | 0.0687 | 95 | 0.364 | 0.7168 |
|  | SR2 v SR3 | 0.02205 | 0.0695 | 95 | 0.317 | 0.7518 |
|  | SR1 v C | -0.05395 | 0.0831 | 95 | -0.649 | 0.518 |
|  | SR2 v C | -0.051 | 0.0813 | 95 | -0.627 | 0.5321 |
|  | SR3 v C | -0.02895 | 0.0989 | 95 | -0.293 | 0.7703 |

**Table S5**: Planned contrasts evaluating the effect of herbivore damage (H1), species identity (H2), and species richness (H3) on fruit quality phenolic content and content by subclass (anthocyanins, flavonols, flavan-3-ols, benzoic acids, dihydrochalcones, and hydroxycinnamic acids). Sf: *Spodoptera frugiperda*; Hv: *Heliothis virescens*; Hz: *Helicoverpa zea*; SR1: Treatments 1, 2, and 3; SR2: Treatments 4, 5, and 6; SR3: Treatment 7.

| **Response Variable** | **Planned Contrasts** | **estimate** | **SE** | ***t*** | ***p*** |
| --- | --- | --- | --- | --- | --- |
| **Phenolic Content** | |  |  |  |  |
|  | ***Overall effects of damage (H1)*** | |  |  |  |
|  | C v D | 0.864 | 0.0872 | -1.445 | 0.1486 |
|  | ***Effects of species identity (H2)*** | |  |  |  |
|  | Sf v Hv | 1.154 | 0.115 | 1.435 | 0.1514 |
|  | Sf v Hz | 1.129 | 0.114 | 1.204 | 0.2285 |
|  | Hv v Hz | 0.978 | 0.101 | -0.216 | 0.8291 |
|  | Sf v C | 0.773 | 0.0943 | -2.109 | 0.035 |
|  | Hv v C | 0.893 | 0.113 | -0.895 | 0.3707 |
|  | Hz v C | 0.873 | 0.0982 | -1.208 | 0.2272 |
|  | ***Effects of herbivore species richness (H3)*** | | |  |  |
|  | SR1 v SR2 | 1.044 | 0.0622 | 0.725 | 0.4687 |
|  | SR1 v SR3 | 1.03 | 0.0891 | 0.338 | 0.7353 |
|  | SR2 v SR3 | 0.986 | 0.0868 | -0.158 | 0.8745 |
|  | SR1 v C | 0.845 | 0.089 | -1.602 | 0.1093 |
|  | SR2 v C | 0.882 | 0.0922 | -1.202 | 0.2293 |
|  | SR3 v C | 0.87 | 0.11 | -1.105 | 0.269 |
| **Anthocyanin Content** | |  |  |  |  |
|  | ***Overall effects of damage (H1)*** | |  |  |  |
|  | C v D | 0.826 | 0.124 | -1.275 | 0.2024 |
|  | ***Effects of species identity (H2)*** | |  |  |  |
|  | Sf v Hv | 0.901 | 0.135 | -0.698 | 0.4851 |
|  | Sf v Hz | 0.82 | 0.126 | -1.292 | 0.1963 |
|  | Hv v Hz | 0.91 | 0.133 | -0.646 | 0.5186 |
|  | Sf v C | 0.887 | 0.17 | -0.627 | 0.5308 |
|  | Hv v C | 0.799 | 0.146 | -1.229 | 0.2189 |
|  | Hz v C | 0.727 | 0.116 | -2.001 | 0.0454 |
|  | ***Effects of herbivore species richness (H3)*** | | |  |  |
|  | SR1 v SR2 | 1.046 | 0.0911 | 0.511 | 0.6095 |
|  | SR1 v SR3 | 1.078 | 0.139 | 0.585 | 0.5585 |
|  | SR2 v SR3 | 1.031 | 0.135 | 0.235 | 0.8139 |
|  | SR1 v C | 0.801 | 0.125 | -1.418 | 0.1562 |
|  | SR2 v C | 0.838 | 0.13 | -1.137 | 0.2557 |
|  | SR3 v C | 0.864 | 0.165 | -0.764 | 0.445 |
| **Flavan-3-ol Content** | |  |  |  |  |
|  | ***Overall effects of damage (H1)*** | |  |  |  |
|  | C v D | 0.826 | 0.112 | -1.405 | 0.16 |
|  | ***Effects of species identity (H2)*** | |  |  |  |
|  | Sf v Hv | 1.318 | 0.173 | 2.1 | 0.0357 |
|  | Sf v Hz | 1.192 | 0.156 | 1.344 | 0.1788 |
|  | Hv v Hz | 0.905 | 0.125 | -0.726 | 0.4677 |
|  | Sf v C | 0.701 | 0.114 | -2.192 | 0.0284 |
|  | Hv v C | 0.924 | 0.159 | -0.458 | 0.6468 |
|  | Hz v C | 0.836 | 0.127 | -1.184 | 0.2363 |
|  | ***Effects of herbivore species richness (H3)*** | | |  |  |
|  | SR1 v SR2 | 1.031 | 0.0808 | 0.386 | 0.6995 |
|  | SR1 v SR3 | 1.002 | 0.114 | 0.021 | 0.9836 |
|  | SR2 v SR3 | 0.972 | 0.112 | -0.243 | 0.808 |
|  | SR1 v C | 0.815 | 0.116 | -1.433 | 0.1519 |
|  | SR2 v C | 0.84 | 0.118 | -1.235 | 0.2167 |
|  | SR3 v C | 0.817 | 0.136 | -1.211 | 0.226 |
| **Flavonol Content** | |  |  |  |  |
|  | ***Overall effects of damage (H1)*** | |  |  |  |
|  | C v D | 1.03 | 0.24 | 0.107 | 0.9147 |
|  | ***Effects of species identity (H2)*** | |  |  |  |
|  | Sf v Hv | 1.78 | 0.411 | 2.494 | 0.0126 |
|  | Sf v Hz | 1.8 | 0.403 | 2.614 | 0.0089 |
|  | Hv v Hz | 1.01 | 0.255 | 0.042 | 0.9665 |
|  | Sf v C | 0.69 | 0.181 | -1.412 | 0.1579 |
|  | Hv v C | 1.23 | 0.374 | 0.675 | 0.4996 |
|  | Hz v C | 1.24 | 0.335 | 0.801 | 0.4234 |
|  | ***Effects of herbivore species richness (H3)*** | | |  |  |
|  | SR1 v SR2 | 1.03 | 0.14 | 0.219 | 0.8268 |
|  | SR1 v SR3 | 0.968 | 0.187 | -0.166 | 0.868 |
|  | SR2 v SR3 | 0.94 | 0.185 | -0.315 | 0.7527 |
|  | SR1 v C | 1.017 | 0.248 | 0.069 | 0.9447 |
|  | SR2 v C | 1.048 | 0.255 | 0.192 | 0.8479 |
|  | SR3 v C | 0.985 | 0.284 | -0.053 | 0.9579 |
| **Benzoic Acid Content** | |  |  |  |  |
|  | ***Overall effects of damage (H1)*** | |  |  |  |
|  | C v D | 0.88 | 0.142 | -0.79 | 0.4297 |
|  | ***Effects of species identity (H2)*** | |  |  |  |
|  | Sf v Hv | 1.24 | 0.198 | 1.345 | 0.1786 |
|  | Sf v Hz | 1.366 | 0.222 | 1.923 | 0.0545 |
|  | Hv v Hz | 1.102 | 0.187 | 0.573 | 0.5668 |
|  | Sf v C | 0.711 | 0.136 | -1.783 | 0.0745 |
|  | Hv v C | 0.882 | 0.181 | -0.612 | 0.5403 |
|  | Hz v C | 0.972 | 0.181 | -0.152 | 0.8796 |
|  | ***Effects of herbivore species richness (H3)*** | | |  |  |
|  | SR1 v SR2 | 1.09 | 0.106 | 0.882 | 0.3778 |
|  | SR1 v SR3 | 1 | 0.139 | -0.002 | 0.9983 |
|  | SR2 v SR3 | 0.917 | 0.13 | -0.606 | 0.5442 |
|  | SR1 v C | 0.848 | 0.144 | -0.972 | 0.3308 |
|  | SR2 v C | 0.924 | 0.156 | -0.466 | 0.6415 |
|  | SR3 v C | 0.848 | 0.172 | -0.815 | 0.4154 |
| **Dihydrochalcone Content** | |  |  |  |  |
|  | ***Overall effects of damage (H1)*** | |  |  |  |
|  | C v D | 0.892 | 0.182 | -0.558 | 0.5767 |
|  | ***Effects of species identity (H2)*** | |  |  |  |
|  | Sf v Hv | 0.998 | 0.208 | -0.011 | 0.9913 |
|  | Sf v Hz | 0.895 | 0.186 | -0.533 | 0.594 |
|  | Hv v Hz | 0.897 | 0.185 | -0.524 | 0.6 |
|  | Sf v C | 0.94 | 0.239 | -0.243 | 0.8076 |
|  | Hv v C | 0.938 | 0.244 | -0.246 | 0.8054 |
|  | Hz v C | 0.842 | 0.192 | -0.755 | 0.4504 |
|  | ***Effects of herbivore species richness (H3)*** | | |  |  |
|  | SR1 v SR2 | 0.953 | 0.115 | -0.403 | 0.6867 |
|  | SR1 v SR3 | 1.044 | 0.185 | 0.241 | 0.8097 |
|  | SR2 v SR3 | 1.096 | 0.198 | 0.507 | 0.6123 |
|  | SR1 v C | 0.905 | 0.196 | -0.458 | 0.6467 |
|  | SR2 v C | 0.862 | 0.179 | -0.711 | 0.4769 |
|  | SR3 v C | 0.945 | 0.246 | -0.217 | 0.8278 |
| **Hydroxycinamic Acid Content** | |  |  |  |  |
|  | ***Overall effects of damage (H1)*** | |  |  |  |
|  | C v D | 1.16 | 0.211 | 0.804 | 0.4215 |
|  | ***Effects of species identity (H2)*** | |  |  |  |
|  | Sf v Hv | 1.16 | 0.218 | 0.803 | 0.4217 |
|  | Sf v Hz | 1.17 | 0.219 | 0.853 | 0.3935 |
|  | Hv v Hz | 1.01 | 0.194 | 0.046 | 0.9634 |
|  | Sf v C | 1 | 0.22 | 0.022 | 0.9827 |
|  | Hv v C | 1.17 | 0.274 | 0.662 | 0.5081 |
|  | Hz v C | 1.18 | 0.253 | 0.766 | 0.4436 |
|  | ***Effects of herbivore species richness (H3)*** | | |  |  |
|  | SR1 v SR2 | 1.072 | 0.121 | 0.616 | 0.5382 |
|  | SR1 v SR3 | 1.06 | 0.174 | 0.356 | 0.7219 |
|  | SR2 v SR3 | 0.989 | 0.165 | -0.069 | 0.9452 |
|  | SR1 v C | 1.114 | 0.217 | 0.557 | 0.5778 |
|  | SR2 v C | 1.195 | 0.225 | 0.946 | 0.3442 |
|  | SR3 v C | 1.181 | 0.271 | 0.725 | 0.4683 |

**Table S6**: Planned contrasts evaluating the effect of herbivore damage (H1), species identity (H2), and species richness (H3) on fruit quality phenolic richness and richness by subclass (anthocyanins, flavonols, flavan-3-ols, benzoic acids, dihydrochalcones, and hydroxycinnamic acids). Sf: *Spodoptera frugiperda*; Hv: *Heliothis virescens*; Hz: *Helicoverpa zea*; SR1: Treatments 1, 2, and 3; SR2: Treatments 4, 5, and 6; SR3: Treatment 7.

| **Response Variable** | **Planned Contrasts** | **estimate** | **SE** | **df** | ***t*** | ***p*** |
| --- | --- | --- | --- | --- | --- | --- |
| **Phenolic Richness** | |  |  |  |  |  |
|  | ***Overall effects of damage (H1)*** | |  |  |  |  |
|  | C v D | -4.79 | 2.02 | 96 | -2.373 | 0.0197 |
|  | ***Effects of species identity (H2)*** | |  |  |  |  |
|  | Sf v Hv | 3.253 | 2.05 | 96 | 1.586 | 0.1161 |
|  | Sf v Hz | 2.577 | 2.09 | 96 | 1.235 | 0.2199 |
|  | Hv v Hz | -0.676 | 2.07 | 96 | -0.326 | 0.7451 |
|  | Sf v C | -7.245 | 2.49 | 96 | -2.912 | 0.0045 |
|  | Hv v C | -3.992 | 2.52 | 96 | -1.581 | 0.1171 |
|  | Hz v C | -4.668 | 2.24 | 96 | -2.088 | 0.0395 |
|  | ***Effects of herbivore species richness (H3)*** | | |  |  |  |
|  | SR1 v SR2 | 1.28 | 1.21 | 96 | 1.061 | 0.2912 |
|  | SR1 v SR3 | -0.365 | 1.75 | 96 | -0.209 | 0.8351 |
|  | SR2 v SR3 | -1.645 | 1.77 | 96 | -0.928 | 0.3558 |
|  | SR1 v C | -5.302 | 2.1 | 96 | -2.52 | 0.0134 |
|  | SR2 v C | -4.022 | 2.07 | 96 | -1.945 | 0.0547 |
|  | SR3 v C | -5.667 | 2.52 | 96 | -2.249 | 0.0268 |
| **Anthocyanin Richness** | |  |  |  |  |  |
|  | ***Overall effects of damage (H1)*** | |  |  |  |  |
|  | C v D | -0.291 | 0.476 | 96 | -0.612 | 0.5418 |
|  | ***Effects of species identity (H2)*** | |  |  |  |  |
|  | Sf v Hv | -0.112 | 0.489 | 96 | -0.23 | 0.8185 |
|  | Sf v Hz | -0.223 | 0.497 | 96 | -0.449 | 0.6544 |
|  | Hv v Hz | -0.111 | 0.494 | 96 | -0.224 | 0.823 |
|  | Sf v C | -0.254 | 0.593 | 96 | -0.429 | 0.6692 |
|  | Hv v C | -0.366 | 0.601 | 96 | -0.609 | 0.5437 |
|  | Hz v C | -0.477 | 0.533 | 96 | -0.896 | 0.3725 |
|  | ***Effects of herbivore species richness (H3)*** | | |  |  |  |
|  | SR1 v SR2 | 0.0342 | 0.287 | 96 | 0.119 | 0.9053 |
|  | SR1 v SR3 | 0.419 | 0.416 | 96 | 1.006 | 0.3168 |
|  | SR2 v SR3 | 0.3848 | 0.422 | 96 | 0.911 | 0.3645 |
|  | SR1 v C | -0.3659 | 0.501 | 96 | -0.73 | 0.4671 |
|  | SR2 v C | -0.3317 | 0.493 | 96 | -0.673 | 0.5024 |
|  | SR3 v C | 0.0531 | 0.6 | 96 | 0.088 | 0.9297 |
| **Flavan-3-ol Richness** | |  |  |  |  |  |
|  | ***Overall effects of damage (H1)*** | |  |  |  |  |
|  | C v D | -2.42 | 1.18 | 96 | -2.052 | 0.0429 |
|  | ***Effects of species identity (H2)*** | |  |  |  |  |
|  | Sf v Hv | 1.629 | 1.21 | 96 | 1.344 | 0.1822 |
|  | Sf v Hz | 0.726 | 1.23 | 96 | 0.589 | 0.5576 |
|  | Hv v Hz | -0.903 | 1.23 | 96 | -0.737 | 0.4629 |
|  | Sf v C | -3.493 | 1.47 | 96 | -2.376 | 0.0195 |
|  | Hv v C | -1.864 | 1.49 | 96 | -1.25 | 0.2145 |
|  | Hz v C | -2.767 | 1.32 | 96 | -2.094 | 0.0389 |
|  | ***Effects of herbivore species richness (H3)*** | | |  |  |  |
|  | SR1 v SR2 | 0.685 | 0.713 | 96 | 0.962 | 0.3386 |
|  | SR1 v SR3 | -0.058 | 1.03 | 96 | -0.056 | 0.9553 |
|  | SR2 v SR3 | -0.743 | 1.05 | 96 | -0.71 | 0.4797 |
|  | SR1 v C | -2.708 | 1.24 | 96 | -2.178 | 0.0318 |
|  | SR2 v C | -2.023 | 1.22 | 96 | -1.655 | 0.1011 |
|  | SR3 v C | -2.766 | 1.49 | 96 | -1.858 | 0.0663 |
| **Flavonol Richness** | |  |  |  |  |  |
|  | ***Overall effects of damage (H1)*** | |  |  |  |  |
|  | C v D | -1.02 | 0.843 | 96 | -1.205 | 0.2312 |
|  | ***Effects of species identity (H2)*** | |  |  |  |  |
|  | Sf v Hv | 1.708 | 0.858 | 96 | 1.99 | 0.0494 |
|  | Sf v Hz | 1.863 | 0.874 | 96 | 2.132 | 0.0355 |
|  | Hv v Hz | 0.155 | 0.868 | 96 | 0.179 | 0.8585 |
|  | Sf v C | -2.211 | 1.05 | 96 | -2.114 | 0.0371 |
|  | Hv v C | -0.503 | 1.06 | 96 | -0.472 | 0.6379 |
|  | Hz v C | -0.348 | 0.943 | 96 | -0.369 | 0.7132 |
|  | ***Effects of herbivore species richness (H3)*** | | |  |  |  |
|  | SR1 v SR2 | 0.25 | 0.503 | 96 | 0.497 | 0.6206 |
|  | SR1 v SR3 | -0.72 | 0.736 | 96 | -0.979 | 0.3301 |
|  | SR2 v SR3 | -0.97 | 0.744 | 96 | -1.304 | 0.1953 |
|  | SR1 v C | -1.02 | 0.888 | 96 | -1.149 | 0.2534 |
|  | SR2 v C | -0.77 | 0.872 | 96 | -0.883 | 0.3793 |
|  | SR3 v C | -1.74 | 1.06 | 96 | -1.64 | 0.1043 |
| **Benzoic Acid Richness** | |  |  |  |  |  |
|  | ***Overall effects of damage (H1)*** | |  |  |  |  |
|  | C v D | -0.394 | 0.31 | 96 | -1.27 | 0.207 |
|  | ***Effects of species identity (H2)*** | |  |  |  |  |
|  | Sf v Hv | 0.2963 | 0.319 | 96 | 0.93 | 0.3547 |
|  | Sf v Hz | 0.7121 | 0.324 | 96 | 2.197 | 0.0304 |
|  | Hv v Hz | 0.4158 | 0.322 | 96 | 1.291 | 0.1997 |
|  | Sf v C | -0.7979 | 0.386 | 96 | -2.065 | 0.0416 |
|  | Hv v C | -0.5016 | 0.392 | 96 | -1.279 | 0.2038 |
|  | Hz v C | -0.0859 | 0.347 | 96 | -0.247 | 0.8053 |
|  | ***Effects of herbivore species richness (H3)*** | | |  |  |  |
|  | SR1 v SR2 | 0.201 | 0.187 | 96 | 1.073 | 0.286 |
|  | SR1 v SR3 | -0.129 | 0.271 | 96 | -0.476 | 0.6354 |
|  | SR2 v SR3 | -0.33 | 0.275 | 96 | -1.199 | 0.2335 |
|  | SR1 v C | -0.462 | 0.327 | 96 | -1.413 | 0.1608 |
|  | SR2 v C | -0.261 | 0.321 | 96 | -0.812 | 0.4186 |
|  | SR3 v C | -0.591 | 0.391 | 96 | -1.51 | 0.1344 |
| **Dihydrochalcone Richness** | |  |  |  |  |  |
|  | ***Overall effects of damage (H1)*** | |  |  |  |  |
|  | C v D | -0.565 | 0.318 | 96 | -1.777 | 0.0787 |
|  | ***Effects of species identity (H2)*** | |  |  |  |  |
|  | Sf v Hv | -0.461 | 0.327 | 96 | -1.413 | 0.161 |
|  | Sf v Hz | -0.768 | 0.332 | 96 | -2.311 | 0.023 |
|  | Hv v Hz | -0.306 | 0.33 | 96 | -0.928 | 0.3557 |
|  | Sf v C | -0.28 | 0.396 | 96 | -0.706 | 0.4817 |
|  | Hv v C | -0.741 | 0.402 | 96 | -1.844 | 0.0683 |
|  | Hz v C | -1.048 | 0.356 | 96 | -2.942 | 0.0041 |
|  | ***Effects of herbivore species richness (H3)*** | | |  |  |  |
|  | SR1 v SR2 | 0.2277 | 0.192 | 96 | 1.186 | 0.2386 |
|  | SR1 v SR3 | 0.1861 | 0.278 | 96 | 0.669 | 0.5053 |
|  | SR2 v SR3 | -0.0416 | 0.282 | 96 | -0.147 | 0.8832 |
|  | SR1 v C | -0.6896 | 0.335 | 96 | -2.058 | 0.0423 |
|  | SR2 v C | -0.4619 | 0.329 | 96 | -1.403 | 0.164 |
|  | SR3 v C | -0.5035 | 0.401 | 96 | -1.255 | 0.2127 |
| **Hydroxycinamic Acid Richness** | |  |  |  |  |  |
|  | ***Overall effects of damage (H1)*** | |  |  |  |  |
|  | C v D | -0.139 | 0.154 | 96 | -0.898 | 0.3714 |
|  | ***Effects of species identity (H2)*** | |  |  |  |  |
|  | Sf v Hv | 0.2 | 0.159 | 96 | 1.261 | 0.2103 |
|  | Sf v Hz | 0.2154 | 0.161 | 96 | 1.335 | 0.1851 |
|  | Hv v Hz | 0.0153 | 0.16 | 96 | 0.096 | 0.9239 |
|  | Sf v C | -0.204 | 0.192 | 96 | -1.06 | 0.2916 |
|  | Hv v C | -0.004 | 0.195 | 96 | -0.021 | 0.9837 |
|  | Hz v C | 0.0113 | 0.173 | 96 | 0.066 | 0.9478 |
|  | ***Effects of herbivore species richness (H3)*** | | |  |  |  |
|  | SR1 v SR2 | -0.12891 | 0.0932 | 96 | -1.383 | 0.17 |
|  | SR1 v SR3 | -0.12541 | 0.135 | 96 | -0.928 | 0.3558 |
|  | SR2 v SR3 | 0.00349 | 0.137 | 96 | 0.025 | 0.9797 |
|  | SR1 v C | -0.06556 | 0.163 | 96 | -0.403 | 0.6878 |
|  | SR2 v C | -0.19446 | 0.16 | 96 | -1.216 | 0.2269 |
|  | SR3 v C | -0.19097 | 0.195 | 96 | -0.98 | 0.3295 |
